# Structure Based docking studies towards exploring potential anti-androgen activity of selected phytochemicals against Prostate Cancer

**DOI:** 10.1038/s41598-017-02023-5

**Published:** 2017-05-16

**Authors:** Anshika N. Singh, Meghna M. Baruah, Neeti Sharma

**Affiliations:** Symbiosis School of Biological Sciences, Symbiosis International University, Gram- Lavale; Taluka - Mulshi, Pune, India

## Abstract

Prostate cancer (PCa) is the second most common malignancy amongst men worldwide. Under PCa maintenance therapy drugs acting as antagonists/partial agonists of hormone receptors against the prostate tissue are used in clinical practices. Prominent drugs being Cyproterone acetate, Flutamide, Bicalutamide, they not only cause acute and long-term toxicity, but also develops drug resistance among patients. Our focus has been on phytochemicals which do not exhibit any cytotoxicity and have significant androgen receptor (AR) inhibition activity. As Protein- Ligand interactions play a key role in structure based drug design, so by using molecular docking, we screened 803 phytochemicals and investigated their binding affinity against AR. The three dimensional (3D) structure of AR was retrieved from Protein Data Bank, and docked with 3D Pubchem structures of 803 phytochemicals using Argus Lab. Molecular docking and drug likeness studies were made using ADMET properties while Lipinski’s rule of five was performed for the phytochemicals to evaluate their anti-prostate cancer activity. The results showed that Isobavachin exhibited best binding affinity of −13.73 kcal/mol with AR followed by Glabranin, Anthocyanin and Eriosemation. Our studies therefore reveal that these four phytochemicals could be promising candidates for further evaluation for PCa prevention or management.

## Introduction

Prostate cancer (PCa) has emerged as the most frequently diagnosed malignancy among men in Western countries^1^. According to current cancer statistics, the total number of reported new cases and deaths from PCa in United States are 180890 and 26120 respectively^2^. The androgen hormones and their executor androgen receptors are known to regulate crucial roles in initiation and progression of PCa^3^. The AR gene is *located* on the X chromosome at the locus *Xq11*-*Xq12* and is activated by binding either of the androgenic hormones, such as testosterone or dihydrotestosterone in the cytoplasm and then translocating into the nucleus^4, 5^. Research has shown that AR contributes to proliferation and differentiation of prostate epithelial cells during development of androgen dependent PCa^6^. Thus over the years, AR has emerged as a potential and attractive target for PCa therapy through application of AR antagonists or combined androgen blockade therapy. The most commonly prescribed AR antagonists for PCa include both steroidal viz. Cyproterone acetate and non- steroidal drugs viz. Flutamide, Bicalutamide, and Enzalutamide^7, 8^. Although a number of gonadotropin releasing hormone (GnRH) agonists and anti-androgens have emerged as the most commonly prescribed chemotherapeutic drugs for PCa the problem of cancer recurrence after short period of response and increased cytotoxicity due to drug intake remains a hurdle in the path of effective therapy. In this context, the positive correlation with phytochemicals intake and reduced risk of PCa has attracted interest and thereby focussed the attention of research towards phytochemicals as chemotherapeutic agents in PCa^9^. These plant compounds which exhibit high binding affinity for prostate cells /androgen receptors could possibly lead to effective PCa treatment.

Phytochemicals are polyphenolic compounds found commonly in food and beverages of plant origin^10^. They are not essential nutrients but have various properties to prevent many diseases including cancer^11^. Structurally they are classified into alkaloids, anthocyanins, carotenoids, coumestans, flavan-3-ols, flavonoids, hydroxycinnamic acids, isoflavones, lignans, monophenols, monoterpenes, organosulfides, phenolic acids, phytosterols, saponins, stylbenes, triterpenoids and xanthophylls^12^. Out of these distinct classes of phytochemicals, flavonoids are most extensively studied compounds and are reported to have a wide range of pharmacological activities such as anti-angiogenic, anti-oxidant, anti-inflammatory, anti-thrombogenic, anti-mutagenic, anti-allergic, anti-bacterial and anti-cancer activity^13, 14^.

The flavonoids are further structurally classified into flavones, flavanols, isoflavones, flavonols, flavanones, flavanonols and anthocyanins^15^. All these subclasses have been reported to arrest cell cycle, induce apoptosis, disrupt mitotic spindle formation and angiogenesis inhibition thus making them potentially versatile anti-cancer agents^15, 16^.

Flavonoids, at molecular level, are found to regulate various protein kinases viz. protein –kinase C, cyclin-dependent kinases and phosphatidylinositol 3-kinases^17^. Studies have shown flavonoids to arrest cell cycle in a dose and time dependent manner and depending on their structure and cancer type, flavonoids can block cell cycle at either G0/G1 or G2/M stage^18, 19^.

One such group of flavonoids, the flavonols have been known to have a structural similarity to testosterone thereby indicating a good possibility of their strong interactions with the AR and significant anti-androgenic activity in PCa^20^. Flavonoids such as quercetin, myricetin, fisetin, morin and kaempferol can inhibit type 1 5-α reductase and hence impairing DHT levels by hampering the testosterone into DHT conversion^21–23^. Studies have shown that these flavonoids can also suppress gene expression of PSA (Prostate Specific Antigen) and KLK2 (Kallikrein) by inhibiting the accumulation of AR in the nucleus^24, 25^. Thus studying the interactions of these versatile flavonoid groups can give insights in their potential application in PCa treatment. Molecular docking is one of such frequently used methods in structure based drug designing because of its ability to predict with a high level of accuracy, the conformation of small-molecule ligands within the appropriate target binding site. Thus it can be employed to study interaction between phytochemicals and AR.

Molecular docking works in a two-step process which starts with compiling different ligand conformations in the identified active site of the receptor and then ranking them according to their binding conformation energies for each individual binding conformation^26^. Various commercially available softwares like Auto Dock, Argus Lab, FLEX, and GOLD are extensively used for estimating docking binding energies of various ligand-receptor conformations^27^. The identification of the most appropriate binding conformations is carried out by exploring the large conformational space depicting various binding sites and then accurately predicting the interaction energy associated with the respective binding conformations^28, 29^. This process is carried out recursively until converging to a solution of minimum energy.

Considering the role of AR in PCa initiation and progression, finding active phytochemical which can block AR activity is an essential step towards development of anti-AR drug. In the present study, we performed molecular docking studies using Argus lab software and evaluated the anti-androgen activity of phytochemicals^30^. Our molecular docking studies are based on the hypothesis that phytochemicals can interfere with androgen activity and result in inhibition of PCa initiation and progression. In the process we have screened 803 phytochemicals which can potentially act as anti-prostate cancer drugs against human AR. The binding affinity of the commonly used drugs for PCa viz., Dihydrotestosterone, Bicalutamide, Abiraterone, Flutamide and Enzalutamide was also evaluated using ArgusLab and was then compared with the binding affinity exhibited by the phytochemicals to come up with a comprehensive list of phytochemicals that have the potential to work as anti-prostate cancer drugs.

## Results and Discussion

### Structure of the target protein

Human androgen receptor (AR) (1E3G) has been exploited as a main therapeutic target for PCa. The three dimensional structure of Human AR was retrieved from the Protein Data Bank with PDB ID: 1E3G.

### Identification of active pockets

The binding site prediction was carried out using Castp to predict three catalytic sites in the receptor molecule with the largest volumes (Table 1)^31^. The first pocket of the AR (PDB ID: 1E3G) has an area of 406.9 and volume of 515.8, second identified pocket has an area of 326.7 and volume of 393.2 and the third active site has an area of 203.6 and volume of 344.2. These identified active sites were used to identify the best ligand binding site.

**Table 1:** Identified active sites of receptor 1E3G with the corresponding interacting residues.

S. No.	Area	Volume	Interacting residues
1	406.9	515.8	*LEU 701, LEU 704, ASN 705, LEU 707, GLY 708, GLN 711, TRP 741, MET 742, MET 745, VAL 746, MET 749, ARG 752, PHE 764, MET 780, MET 787, LEU 873, PHE 876, THR 877, LEU 880, MET 895, ILE 899*
2	326.7	393.2	*GLU 681, PRO 682, GLY 683, VAL 684, VAL 685, GLN 711, HIS 714, VAL 715, LEU 744, MET 745, ALA 748, TRP 751, ARG 752, ASN 756, TYR 763, PHE 764, ALA 765, PRO 766, PHE 804, LYS 808*
3	203.6	344.2	*GLN 798, THR 800, GLU 803, ILE 841, ILE 842, LYS 845, ARG 846, LYS 847, SER 851, CYS 852, ARG 855*

### Molecular docking using Argus Lab 4.0.1 and Drug likeliness

**Figure 1:**
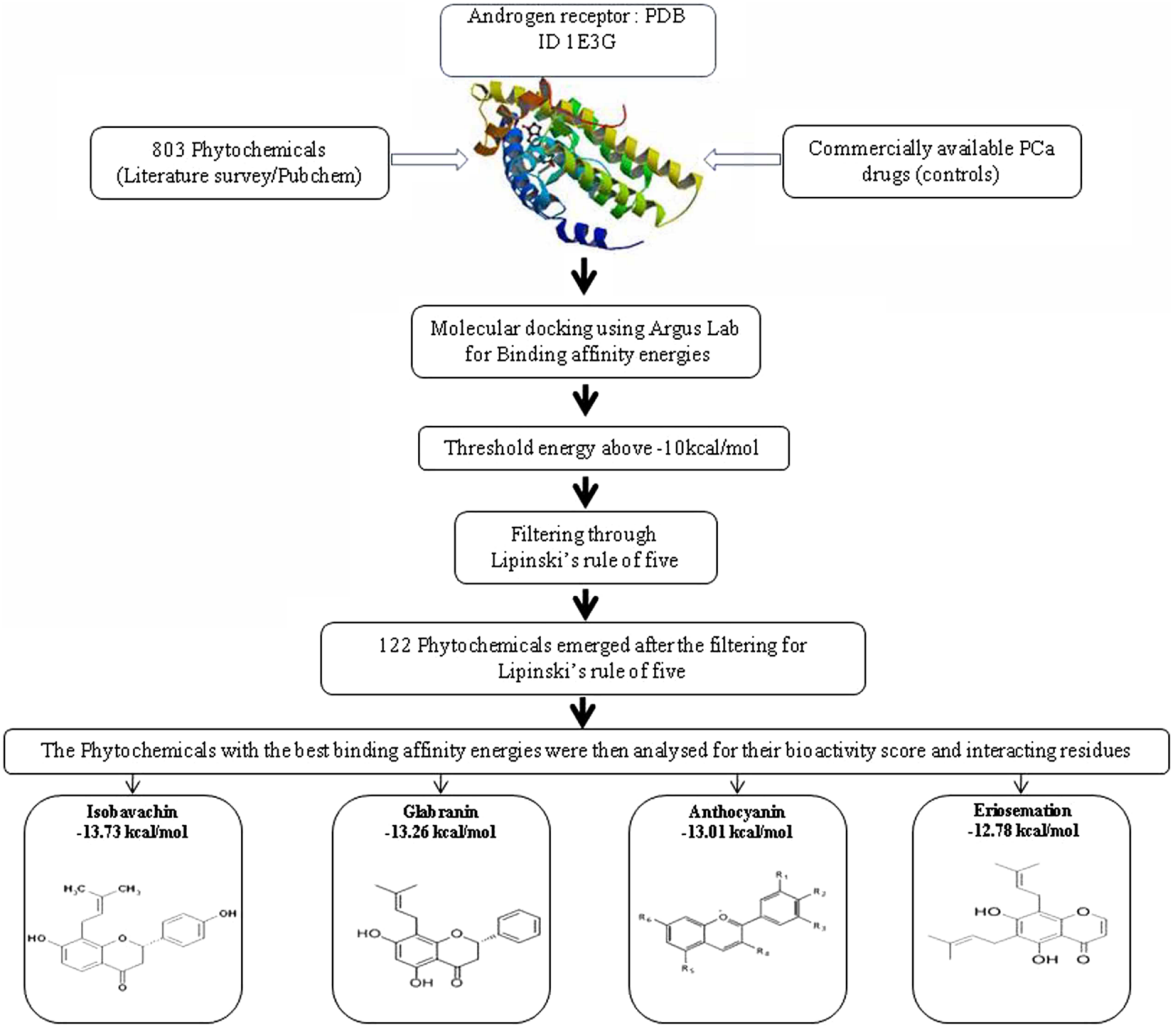
Graphical representation of the different approaches used in this study.

During the initial part of the study we docked various commercially available PCa drugs in the identified binding sites to draw a comparison with the binding energy of the phytochemicals (Fig. 1). Commercially available PCa drugs are used as controls in the study. The threshold values were then decided with respect to binding affinity energies of controls (Table 2).

**Table 2:** Binding affinity energies and the interacting catalytic sites of the commercially available PCa drugs.

Commercially available drugs for PCa	Structure	Binding affinity (kcal/mol)	Interacting residues
Dihydrotestosterone		−13.92 kcal/mol	First pocket
Bicalutamide		−11.83 kcal/mol	First pocket
Abiraterone		−10.32 kcal/mol	Second pocket
Flutamide		−8.69 kcal/mol	First pocket
Enzalutamide		−8.10 kcal/mol	Second pocket

The ligand conformations of 803 phytochemicals were then ranked according to their binding affinities with AR (Supplementary Table S1). Our study revealed that among the commercially available PCa drugs, the best docking energy was exhibited by Dihydrotestosterone (DHT) with binding affinity of −13.92 kcal/mol followed by Bicalutamide (−11.83 kcal/mol) and Abiraterone (−10.32 kcal/mol) (Table 2).

**Table 3:** Binding affinity energy of best docking pose of the shortlisted phytochemicals against 1E3G.

Phytochemicals	Chemical structure	Binding affinity energy
Isobavachin		−13.73 kcal/mol
Glabranin		−13.26 kcal/mol
Anthocyanin		−13.01 kcal/mol
Eriosemation		−12.78 kcal/mol

Amongst the 803 phytochemicals screened, 122 phytochemicals exhibited docking energies values above −10 kcal/mol (Supplementary Table S2) which were close to the one exhibited by Abiraterone (−10.32 kcal/mol) and thus these 122 phytochemicals were selected for further evaluation as they exhibited docking energy of greater than −10 kcal/mol. Molecular properties of all these 122 phytochemicals were then investigated using Molinspiration software to satisfy Lipinski’s rule of five, which is essential for rational drug design and also to determine their bioactivity score^32^. It was found that the selected 122 phytochemicals showed no violation of all the five rules i.e. not more than 5 hydrogen bond donors, not more than 10 hydrogen bond acceptors, molecular weight of compounds less than 500, partition coefficient (log P) less than 5, rotatable bonds less than 10, topological polar surface area (TPSA) of not greater than 140.

Subsequently for further analysis the top four, phytochemicals with highest binding affinity energies viz. Isobavachin, Glabranin, Anthocyanin and Eriosemation were used. In these top four evaluations, it was found that Isobavachin showed the best docking conformation with binding affinity of −13.73 kcal/mol with AR closely followed by Glabranin, Anthocyanin and Eriosemation with binding affinities of −13.26 kcal/mol, −13.01 kcal/mol and −12.78 kcal/mol respectively (Supplementary Figure 1) (Table 3).

These four phytochemicals which belong to the flavonoid group viz. Isobavachin, Glabranin, Anthocyanin and Eriosemation exhibit good bioactivity properties and drug-likeliness properties as presented in Table 4a and b. They were then further analysed to identify their best docking pose. Molecular docking revealed that the most interacting residues in the active site of AR included Leu 873, Gly 708, Arg 752, Met 745, Gln 711 and Met 749. Isobavachin showed interaction with Leu 873, Gly 708, Arg 752, Met 745, Glabranin was seen to interact with Gly 708, Gln 711, Arg 752, Met 745, Met 749 and Anthocyanin interacted with Asp 879, Leu 880, Met 780, Trp 741, Leu 873, Met 787, Met 895, Arg 752, Gln 711 while Eriosemation interacted with Thr 877 and Leu 873 respectively (Table 5).

**Table 4:** (**a**) The Lipinski’s rule of five attributes of shortlisted phytochemicals.

Phytochemicals	miLogP	TPSA	natoms	MW	nON	nOHNH	nviolation	nrotb	volume
Isobavachin	4.45	66.76	24	324.38	4	2	0	3	299.58
Glabranin	4.85	66.76	24	324.38	4	2	0	3	299.58
Anthocyanin	1.31	11.17	16	207.25	1	0	0	1	194.72
Eriosemation	4.85	70.67	23	314.38	4	2	0	4	299.30
**Phytochemicals**	**GPCR ligand**	**Ion channel modulator**	**Kinase inhibitor**	**Nuclear receptor ligand**	**Protease inhibitor**	**Enzyme inhibitor**			
Isobavachin	0.26	−0.13	−0.18	0.72	0.09	0.44			
Glabranin	0.22	−0.05	−0.15	0.72	0.11	0.45			
Anthocyanin	−0.61	−0.30	−0.57	−0.65	−0.75	−0.38			
Eriosemation	0.11	−0.11	−0.23	0.42	−0.24	0.48			

(**b**) Molinspiration bioactivity score of shortlisted phytochemicals.

**Table 5:** Intermolecular H bonds between shortlisted phytochemicals with 1E3G.

Phytochemicals	Interacting residues	Distance
Isobavachin	Atom (4112 O) in residue (253 no residue name) H bonds with Atom (3350 O) in residue (873 Leu)	2.51 Å
	Atom (569 N) in residue (708 Gly) H bonds with Atom (4110 O) in residue (253 no residue name)	2.33 Å
	Atom (1294 N) in residue (752 Arg) H bonds with Atom (4110 O) in residue (253 no residue name)	2.99 Å
	Atom (4110 O) in residue (253 no residue name) H bonds with (1176 O) in residue (745 Met)	2.60 Å
Glabranin	Atom (569 N) in residue (708 Gly) H bonds with Atom (4110 O) in residue (253 no residue name)	2.99 Å
	Atom (4110 O) in residue (253 no residue name) with Atom (622 O) in residue (711 Gln)	2.85 Å
	Atom (623 N) in residue (711 Gln) H bonds with Atom (4111 O) in residue (253 no residue name)	2.35 Å
	Atom (1294 N) in residue (752 Arg) H bonds with Atom (4111 O) in residue (253 no residue name)	2.74 Å
	Atom (4112 O) in residue (253 no residue name) H bonds with Atom (1176 O) in residue (745 Met)	1.96 Å
	Atom (1236 N) in residue (749 Met) H bonds with Atom (4112 O) in residue (253 no residue name)	2.72 Å
Anthocyanin	Atom (1735 N) in residue (880 Leu) bonds with Atom (1730 O) in residue (879 Asp)	2.24 Å
	Atom (1768 N) in residue (884 Ser) H bonds with Atom (1738 O) in residue (880 Leu)	2.74 Å
Eriosemation	Atom (4111 O) in residue (253 no residue name) H bonds with (3425 O) in residue (877 Thr)	2.99 Å
	Atom (4111 O) in residue (253 no residue name) H bonds with Atom (3850 O) in residue (873 Leu)	2.89 Å
	Atom (4112 O) in residue (253 no residue name) H bonds with Atom (3350 O) in residue (873 Leu)	2.44 Å

Therefore in the current study Isobavachin, Glabranin, Anthocyanin and Eriosemation emerged as prospective drug candidates that can be further evaluated and applied against androgen receptor for treatment of PCa. Isobavachin is a flavanone^33^ with a prenyl group at position 8 of ring A. Studies have shown that Isobavachin (IBA) facilitates differentiation of mouse embryonic cells into embryonic cells through protein prenylation^34^. IBA has also been shown to regulate phos-ERK activation and phos-p38 off pathway which are known to be dysregulated in various cancers^34^. IBA is also reported to be used as regulator of estrogen receptors ERalpha36 for treatment of tumours, osteoporosis, heart diseases, asthma and senile dementia^35^.

Glabranin, a flavanone (sub class of flavonoids) derived from Glycyrrhiza glabra L. leaves is well known for its antioxidant, anti-genotoxic and anti- inflammatory activities and various patents have been claimed for its role in stimulating hair growth and cosmetics^36–40^. Glabranin fulfils all the drug likeness and ADMET (absorption, distribution, metabolism, excretion and toxicity) properties and emerges as novel potent anti-chemotherapeutic agent in our analysis.

Anthocyanins are the most abundant flavonoids found naturally in fruits and vegetables^41^. Anthocyanins occur as glycosides having galactose rhamnose, xylose arabinose or glucose attached to aglycone nucleus^42, 43^.These are extensively studied *in vitro* for their anti- cancer properties, antioxidants, phase II enzyme activation, anti-proliferation activity, induction of apoptosis, anti- angiogenesis, anti- invasiveness and also for induction of differentiation^41, 43^. *In vitro* studies have also shown that Anthocyanins regulates cancer initiation and development^44^. Several *in vivo* studies reported in oesophageal cancer, colorectal cancer, skin cancer and lung cancer have shown positive chemotherapeutic activities of Anthocyanins^45–48^.

Eriosemation is derived from the roots of *Lupinus luteus* ^49^ and is relatively unexplored for its biological activities to the best of our knowledge. Our study is novel and significant in the sense that it highlights importance of Eriosemation as a potential chemotherapeutic agent against PCa based on its good binding affinity energy with AR.

In pharmaceutical research, computational strategies are of great value as they help in the identification and development of novel promising compounds especially by molecular docking methods^27, 29^. Various research groups have applied these methods to screen potential novel compounds against a variety of diseases^28^. Recently, by employing similar approaches a group of researchers identified natural compounds viz. Chrysin and Equol exhibiting anti- breast cancer activities^50^. In the present study we have explored and simultaneously demonstrated that among 803 phytochemicals screened 122 phytochemicals exhibited anti- androgen activity and majority of these belonged to the class of flavonoids. However, Isobavachin showed the best binding affinity energy of −13.73 kcal/mol with the AR followed by Glabranin, Anthocyanins and Eriosemation. This is the first study to the best of our knowledge to highlight the importance of Eriosemation as a potential chemotherapeutic agent for PCa besides Isobavachin, Glabranin and Anthocyanins.

On the whole, we conclude that the four identified flavonoids viz. Isobavachin, Glabranin, Anthocyanin and Eriosemation have the desired qualities to be potent anti-prostate cancer drugs against Human AR. Thus it is worth to carry further investigations at both *in vitro* and *in vivo* levels on them to be applied for prevention and/or management of PCa.

## Methods

### Selection of receptor and ligands

Majority of the phytochemicals (803) reported were considered for molecular docking study (https://pubchem.ncbi.nlm.nih.gov/ and http://www.chemfaces.com/compound/ Phytochemicals.php). Dihydrotestosterone and commercially available PCa drugs such as Bicalutamide, Abiraterone, Flutamide and Enzalutamide were considered as controls for the study.

### Protein preparation

The protein “androgen receptor (AR)” was prepared by retrieving the three-dimensional crystal structure of Human AR ligand binding in complex with ligand metribolone (PDB: 1E3G) from RCSB PDB (http://www.rcsb.org/pdb/home/home.do). The protein was subsequently cleaned by removing the bound complex molecule, the non-essential water molecules and all the heteroatoms. Finally, hydrogen atoms were merged to the receptor molecule in Argus Lab. The three main catalytic sites of the receptor molecule 1E3G were identified using Computed Atlas of Surface Topography of Proteins (Castp) program (http://sts.bioe.uic.edu/castp/).

### Ligand preparation

The three dimensional structures of shortlisted 803 phytochemicals known to have anti-cancer activities and controls (DHT and commercially prescribed drugs) were downloaded from Pubchem (https://pubchem.ncbi.nlm.nih.gov/) in SDF format. The SDF files were then converted to PDB format using Open Babel Convertor (http://openbabel.org/wiki/Main_Page) and further used for molecular docking analysis.

### Molecular docking using ArgusLab

Subsequent to receptor and ligand preparation, molecular docking analysis was performed by ArgusLab 4.0.1 (shape based search algorithm) to evaluate the hydrogen bond interaction and their binding affinities. For our analysis we used the “Argus Dock” exhaustive search docking function at a regular precision and flexible docking mode. After the minimisation process, the grid box resolution was set at 18 × 18 × 20 Å along the x, y and z axes respectively at grid resolution of 0.40 Å to define the binding site. The controls were first docked with the binding site of “AR” and the resulting interactions were compared with those calculated docking results of shortlisted 803 phytochemicals into the similar active sites using the same grid box dimension.

### Drug likeness calculations

Drugs scans were carried out to determine whether the phytochemicals fulfil the drug-likeness conditions. Lipinski’s filters using Molinspiration (http://www.molinspiration.com/) were applied for examining drug likeness attributes as including quantity of hydrogen acceptors (should not be more than 10), quantity of hydrogen donors (should not be more than 5), molecular weight (mass should be more than 500 daltons) and partition coefficient log P (should not be less than 5). The smiles format of each of the phytochemical was uploaded for the analysis.

## Electronic supplementary material

Supplementary Information

## References

[1] Siegel RL, Miller KD, Jemal A (2016). Cancer statistics, 2016. CA Cancer J. Clin..

[2] National Cancer Institute at National Institute of Health. *SEER Stat Fact Sheets: Prostate Cancer* http://seer.cancer.gov/statfacts/html/prost.html (2015).

[3] Suzuki H, Ueda T, Ichikawa T, Ito H (2003). Androgen receptor involvement in the progression of prostate cancer. Endocr. Relat Cancer.

[4] Tan MH, Li J, Xu HE, Melcher K, Yong EL (2015). Androgen receptor: structure, role in prostate cancer and drug discovery. Acta Pharmacol. Sin..

[5] Dehm SM, Tindall DJ (2006). Molecular regulation of androgen action in prostate cancer. J. Cell Biochem..

[6] Singh M (2014). Stromal androgen receptor in prostate development and cancer. Am. J. Pathol..

[7] Goldenberg SL, Bruchovsky N (1991). Use of cyproterone acetate in prostate cancer. Urol. Clin. North Am..

[8] Gao W, Kim J, Dalton JT (2006). Pharmacokinetics and pharmacodynamics of nonsteroidal androgen receptor ligands. Pharm. Res..

[9] Bommareddy A (2013). Chemoprevention of prostate cancer by major dietary phytochemicals. Anticancer research.

[10] Doughari, J. H., Human, I. S., Benadé, A. J. S. & Ndakidemi, P. A. Phytochemicals as chemotherapeutic agents and antioxidants: Possible solution to the control of antibiotic resistant verocytotoxin producing bacteria. http://hdl.handle.net/11189/2367. (2009)

[11] Rao BN (2003). Bioactive phytochemicals in Indian foods and their potential in health promotion and disease prevention. Asia Pacific Journal of clinical nutrition.

[12] Top Cultures. Phytochemicals. http://www.phytochemicals.info/phytochemicals.php (2016).

[13] Kumar S, Pandey A (2013). Chemistry and Biological Activities of Flavonoids: An Overview. The Scientific World Journal.

[14] Chahar MK, Sharma N, Dobhal MP, Joshi YC (2011). Flavonoids: A versatile source of anticancer drugs. Pharmacogn. Rev..

[15] Tazzini, N. Flavonoids: Definition, Structure and Classification. http://www.tuscany-diet.net/2014/01/22/flavonoids-definition-structure-classification/ (2014).

[16] Batra P, Sharma A (2013). Anti-cancer potential of flavonoids: recent trends and future perspectives. 3 Biotech.

[17] Hou D, Kumamoto T (2010). Flavonoids as Protein Kinase Inhibitors for Cancer Chemoprevention: Direct Binding and Molecular Modeling. Antioxid. Redox Signal..

[18] Woo HH, Jeong BR, Hawes MC (2005). Flavonoids: from cell cycle regulation to biotechnology. Biotechnol. Lett..

[19] Kobayashi T, Nakata T, Kuzumaki T (2002). Effect of flavonoids on cell cycle progression in prostate cancer cells. Cancer Lett..

[20] Boam T (2015). Anti-androgenic effects of flavonols in prostate cancer. Ecancermedicalscience..

[21] Hiipakka RA, Zhang HZ, Dai W, Dai Q, Liao S (2002). Structure-activity relationships for inhibition of human 5alpha-reductases by polyphenols. Biochem. Pharmacol..

[22] Baruah MM, Sharma N, Khandwekar AP (2016). Flavonoids and Prostate Cancer. AIJRFANS.

[23] Zand RSR, Jenkins DJ, Brown TJ, Diamandis EP (2002). Flavonoids can block PSA production by breast and prostate cancer cell lines. Clinica chimica acta.

[24] Yuan H, Gong A, Young CY (2005). Involvement of transcription factor Sp1 in quercetin-mediated inhibitory effect on the androgen receptor in human prostate cancer cells. Carcinogenesis.

[25] Vue B, Zhang S, Chen QH (2016). Flavonoids with Therapeutic Potential in Prostate Cancer. Anticancer Agents Med. Chem.

[26] Seyedi SS (2016). Corrigendum: Computational Approach Towards Exploring Potential Anti-Chikungunya Activity of Selected Flavonoids. Sci. Rep..

[27] Lounnas V (2013). Current progress in Structure-Based Rational Drug Design marks a new mindset in drug discovery. Comput. Struct. Biotechnol. J..

[28] Ferreira LG, Dos Santos RN, Oliva G, Andricopulo AD (2015). Molecular docking and structure-based drug design strategies. Molecules..

[29] Yuriev E, Ramsland PA (2013). Latest developments in molecular docking: 2010–2011 in review. J. Mol. Recognit.

[30] Thompson MA (2004). Molecular docking using ArgusLab, an efficient shape-based search algorithm and the AScore scoring function. Proceedings of the ACS meeting Philadelphia.

[31] Dundas J (2006). CASTp: computed atlas of surface topography of proteins with structural and topographical mapping of functionally annotated residues. Nucleic acids research.

[32] Molinspiration Cheminformatics. *Molinspiration* http://www.molinspiration.com/cgi-bin/properties (2016).

[33] Azimova, A. V. & Vinogradova, V. I. Isobavachin. Natural compounds. *Springer New York*. https://link.springer.com/referenceworkentry/10.1007%2F978-1-4614-0535-1_564 (2013).

[34] Wang DY (2011). Promoting effects of isobavachin on neurogenesis of mouse embryonic stem cells were associated with protein prenylation. Acta Pharmacol. Sin..

[35] Jing, L. & Kun, M. Applications of Isobavachin for preparing antineoplastic, osteoporosis and senile dementia medicament (PAT-CN101249089) (2008).

[36] Shinde DB, Koratkar SS, Sharma N, Shitole AA (2016). Antioxidant activity and antiproliferative action of methanolic extract of liquorice (*Glycyrrhiza glabra*) in HepG2 cell line. Int. J. Pharm. Pharm.Sci..

[37] Biondi DM, Rocco C, Ruberto G (2005). Dihydrostilbene derivatives from Glycyrrhiza glabra leaves. J. Nat. Prod..

[38] Siracusa L (2011). Phytocomplexes from liquorice (Glycyrrhiza glabra L.) leaves–chemical characterization and evaluation of their antioxidant, anti-genotoxic and anti-inflammatory activity. Fitoterapia.

[39] Bhogal, R. K. & Rogers, J. S. Use of glabranin for stimulating hair growth (PAT- US20130096189 A1) (2013).

[40] Szaloki, E., Kristof, I., Pal, V. & Szabo, E. Cosmetic composition. (PAT- US5468492A) (1993).

[41] Wang LS, Stoner GD (2008). Anthocyanins and their role in cancer prevention. Cancer Lett..

[42] Mazza G (1995). Anthocyanins in grapes and grape products. Crit Rev. Food Sci. Nutr..

[43] Hou DX, Fujii M, Terahara N, Yoshimoto M (2004). Molecular mechanisms behind the chemopreventive effects of anthocyanidins. BioMed Research International.

[44] Wang, L. S. *et al*. Anthocyanins and cancer prevention In *Nutraceuticals and Cancer* 201–229 (Springer, Netherlands, 2012).

[45] Peiffer DS (2014). Chemoprevention of esophageal cancer with black raspberries, their component anthocyanins, and a major anthocyanin metabolite, protocatechuic acid. Cancer Prev. Res. (Phila).

[46] Thomasset S (2009). Pilot study of oral anthocyanins for colorectal cancer chemoprevention. Cancer Prev. Res. (Phila).

[47] Afaq F (2007). Delphinidin, an anthocyanidin in pigmented fruits and vegetables, protects human HaCaT keratinocytes and mouse skin against UVB-mediated oxidative stress and apoptosis. J. Invest Dermatol..

[48] Al-Jasabi S, Saad A, Haque EE (2013). The Role of Antioxidant Anthocyanin in the Attenuation of Lung Cancer Caused by Benzo [A] Pyrene in Balb/C Mice. Middle-East J. Sci. Res.

[49] Chemfaces. http://www.chemfaces.com/natural/Eriosemation-CFN92835.html (2016).

[50] Suganya J, Radha M, Naorem DL, Nishandhini M (2014). In Silico docking studies of selected flavonoids–natural healing agents against breast cancer. Asian Pac. J. Cancer Prev..

