# Supplementary material for: Structure Based docking studies towards exploring potential anti-androgen activity of selected phytochemicals against Prostate Cancer
Source: Sci Rep. 2017 May 16;7:1955. doi: 10.1038/s41598-017-02023-5 (PMC5434041; doi:10.1038/s41598-017-02023-5)

Title: Structure Based docking studies towards exploring potential anti-androgen activity of selected phytochemicals against Prostate Cancer

Anshika N. Singh[#], Meghna M. Baruah[#], and Neeti Sharma*
Symbiosis School of Biomedical Sciences, Symbiosis International University, Gram- Lavale; Taluka - Mulshi, Pune, India.

([#] Combined first author)

,

Supplementary Table S1: Binding affinity of 803 phytochemicals with Androgen receptor (Note: The phytochemicals were checked for their binding affinities first in the first followed by second and finally in the third active site pocket. phytochemicals exhibiting binding affinity in the 1st pocket were not evaluated for the 2nd and 3rd pocket and similarly flavonoids exhibiting binding energy in the 2nd pocket were not evaluated for the 3rd pocket).

S.NO	PHYTOCHEMICALS	FIRST ACTIVE SITE	SECOND ACTIVE SITE	THIRD ACTIVE SITE
1	Neoisoliquiritin	No binding affinity	-9.51875	-
2	Alcesefoliside	No binding affinity	No binding affinity	No binding affinity
3	Mauritianin	No binding affinity	No binding affinity	No binding affinity
4	Semilicoisoflavone B	No binding affinity	No binding affinity	No binding affinity
5	Glycyrrhisoflavone	No binding affinity	No binding affinity	No binding affinity
6	Licoisoflavone A	No binding affinity	-8.07268	-
7	Isosinensetin	No binding affinity	No binding affinity	No binding affinity
8	Pratensein	No binding affinity	No binding affinity	No binding affinity
9	Vitexin argininate	No binding affinity	-10.3039	-
10	Isoanhydroicaritin	No binding affinity	No binding affinity	No binding affinity
11	5-O-Demethylnobiletin	No binding affinity	-8.21469	-
12	Luteolin-3-O-beta-D-glucuronide	No binding affinity	No binding affinity	No binding affinity
13	Kushenol F	No binding affinity	-11.23	-
14	Quercetin-3-gentiobioside	No binding affinity	No binding affinity	-9.29663
15	2''-O-Beta-L-Galorientin	No binding affinity	No binding affinity	No binding affinity
16	Quercetin Dihydrate	No binding affinity	No binding affinity	-9.19611
17	5,7,3'-Trihydroxy-6,4',5'-trimethoxyflavone	-9.31894	-	-
18	Isomucronulatol 7-O-glucoside	No binding affinity	-8.0793	-
19	6-Demethoxytangeretin	No binding affinity	No binding affinity	No binding affinity
20	Camelliaside B	No binding affinity	No binding affinity	No binding affinity
21	Sotetsuflavone	No binding affinity	No binding affinity	No binding affinity

22	Gardenin B	No binding affinity	No binding affinity	-7.23453
23	Isoliquiritin apioside	- No binding affinity	No binding affinity	-6.30097
24	6''-O-Acetylglycitin	No binding affinity	-8.3721	-
25	Camelliaside A	No binding affinity	No binding affinity	No binding affinity
26	Irigenin	No binding affinity	-7.24875	-
27	Troxerutin	No binding affinity	No binding affinity	No binding affinity
28	Dihydrobaicalein	No binding affinity	No binding affinity	No binding affinity
29	6,7,4'-Trihydroxyisoflavone	No binding affinity	No binding affinity	No binding affinity
30	Chrysoeriol-7-O-glucoside	No binding affinity	-8.44134	-
31	Tectorigenin 7-O-xylosylglucoside	No binding affinity	No binding affinity	-7.19422
32	4'-Methoxypuerarin	No binding affinity	-7.95156	-
33	Kaempferol 3,7,4'-trimethylether	-9.34076	-	-
34	Boeravinone E	No binding affinity	-8.90729	-
35	3'-Methoxydaidzein	No binding affinity	-9.4442	-
36	Demethylvestitol	-10.3749	-	-
37	3'-Methoxypuerarin	No binding affinity	-8.0959	-
38	Isoxanthohumol	-11.7745	-	-
39	Sophoflavescenol	No binding affinity	-9.00729	-
40	Isokurarinone	No binding affinity	-10.0199	-
41	Baohuoside VII	No binding affinity	No binding affinity	No binding affinity
42	Baohuoside II	No binding affinity	-10.3787	-
43	Baohuoside V	No binding affinity	No binding affinity	No binding affinity
44	Epimedoside A	No binding affinity	No binding affinity	No binding affinity
45	Mirabijalone D	No binding affinity	No binding affinity	No binding affinity
46	1''-Hydroxyerythrinin C	No binding affinity	No binding affinity	No binding affinity
47	9-O-Methyl-4-hydroxyboeravinone B	No binding affinity	No binding affinity	No binding affinity
48	2,3-Dihydroamentoflavone 7,4'-dimethyl ether	No binding affinity	No binding affinity	No binding affinity
49	Ayanin	-8.705254	-	-

50	Ombuin 3-glucoside	No binding affinity	-7.5556	-
51	Dihydrodaidzin	No binding affinity	No binding affinity	No binding affinity
52	Lupiwighteone	No binding affinity	-9.82408	-
53	Pyrroside B	No binding affinity	No binding affinity	No binding affinity
54	2',7-Dihydroxy-5,8-dimethoxyflavanone	No binding affinity	No binding affinity	No binding affinity
55	2,3-Dihydroisoginkgetin	No binding affinity	No binding affinity	No binding affinity
56	2,3-dihydrosciadopitysin	No binding affinity	No binding affinity	No binding affinity
57	Tsugafolin	No binding affinity	-8.61081	-
58	Thevetiaflavone	No binding affinity	-8.40172	-
59	2,3-Dihydroheveaflavone	No binding affinity	No binding affinity	No binding affinity
60	Isorhamnetin 3-glucoside-7-rhamnoside	No binding affinity	-6.99861	-
61	Quercetin 3-O-glucoside-7-O-rhamnoside	No binding affinity	No binding affinity	No binding affinity
62	Vincetoxicoside B	No binding affinity	No binding affinity	No binding affinity
63	Kaempferol 3-sophoroside-7-rhamnoside	No binding affinity	No binding affinity	No binding affinity
64	Isorhamnetin 3-sophoroside-7-rhamnoside	No binding affinity	No binding affinity	No binding affinity
65	6-Aldehydoisoophiopogonanone A	No binding affinity	-8.39591	-
66	4',7-Isoflavandiol	No binding affinity	No binding affinity	No binding affinity
67	Carlinoside	No binding affinity	No binding affinity	No binding affinity
68	Quercetin 3-O-beta-D-xylopyranoside	No binding affinity	-10.3061	-
69	Aloeresin D	No binding affinity	-9.45539	-
70	7-O-Methylaloeresin A	No binding affinity	-9.99347	-
71	5,7,4'-Trihydroxy-8-methylflavanone	No binding affinity	No binding affinity	No binding affinity
72	Liquiritin apioside	No binding affinity	No binding affinity	-9.76382
73	Methylophiopogonone A	No binding affinity	No binding affinity	No binding affinity
74	Kaempferol-3-O-glucorhamnoside	No binding affinity	No binding affinity	No binding affinity
75	Scutellarin methylester	No binding affinity	-8.73684	-
76	Dihydrodaidzein	-11.5459	-	-
77	7''-O-Methylsciadopitysin	No binding affinity	No binding affinity	No binding affinity

78	5-Dehydroxyparatocarpin K	No binding affinity	-9.255	-
79	6-Methyl-7-O-methylaromadendrin	No binding affinity	No binding affinity	No binding affinity
80	3'-Hydroxypuerarin	No binding affinity	-8.4357	-
81	Apigenin 7-O-(2G-rhamnosyl)gentiobioside	No binding affinity	No binding affinity	No binding affinity
82	6'''-Feruloylspinosin	No binding affinity	No binding affinity	No binding affinity
83	Vitexin 2''-O-p-coumarate	No binding affinity	No binding affinity	No binding affinity
84	Orientin 2''-O-p-trans-coumarate	No binding affinity	No binding affinity	No binding affinity
85	Swertiajaponin	No binding affinity	-7.19113	-
86	Isokaempferide	-9.94241	-	-
87	Hispidulin 7-O-neohesperidoside	No binding affinity	No binding affinity	No binding affinity
88	Mirificin	No binding affinity	No binding affinity	-7.8718
89	Puerarin 6''-O-xyloside	No binding affinity	No binding affinity	No binding affinity
90	6''-O-Malonylgenistin	No binding affinity	-9.99348	-
91	Quercetin-3-O-sophoroside	No binding affinity	No binding affinity	No binding affinity
92	Swertisin	No binding affinity	No binding affinity	No binding affinity
93	Dehydrotoxicarol	No binding affinity	-7.5275	-
94	Dihydroobovatin	No binding affinity	No binding affinity	No binding affinity
95	Erysenegalensein E	No binding affinity	-10.1676	-
96	Isoerysenegalensein E	No binding affinity	-10.7655	-
97	Auriculasin	No binding affinity	-9.16629	-
98	Syzalterin	No binding affinity	No binding affinity	No binding affinity
99	1''-Methoxyerythrinin C	No binding affinity	No binding affinity	No binding affinity
100	Angophorol	No binding affinity	-8.18296	-
101	Phaseollidin hydrate	No binding affinity	-9.20195	-
102	8-Lavandulylkaempferol	No binding affinity	No binding affinity	No binding affinity
103	Derrisisoflavone B	No binding affinity	-12.7182	-
104	Pedalitin	No binding affinity	No binding affinity	No binding affinity
105	5,7,4'-Tri-O-methylcatechin	No binding affinity	-7.13996	-

106	7-O-Methylporiol	No binding affinity	-8.11829	-
107	Dodoviscin J	No binding affinity	No binding affinity	No binding affinity
108	Kuwanol C	No binding affinity	-9.40904	-
109	Isorhamnetin 3-glucuronide	No binding affinity	-7.50418	-
110	Hesperetin 5-O-glucoside	No binding affinity	-6.88324	-
111	Dodoviscin A	No binding affinity	-6.78908	-
112	Dodoviscin I	No binding affinity	-10.2984	-
113	Aliarin	No binding affinity	-9.52652	-
114	Dodoviscin H	No binding affinity	-9.68899	-
115	11-Hydroxytephrosin	No binding affinity	-6.80264	-
116	5,7,4-Trihydroxy-3,6-dimethoxy-3-prenylflavone	No binding affinity	No binding affinity	No binding affinity
117	Bidwillol A	No binding affinity	-11.0226	-
118	Atalantoflavone	No binding affinity	-8.79933	-
119	Neorauflavene	No binding affinity	-8.87879	-
120	Sanggenon N	No binding affinity	-10.1305	-
121	Glepidotin B	-12.0186	-	-
122	3,6-Dimethoxyapigenin	No binding affinity	No binding affinity	No binding affinity
123	5'-Prenylaliarin	No binding affinity	No binding affinity	No binding affinity
124	4'-O-Methyllicoflavanone	No binding affinity	-9.27829	-
125	2-Hydroxynaringenin	-10.6282	-	-
126	Maltol	-7.04116	-	-
127	2''-Acetylastragalin	No binding affinity	No binding affinity	No binding affinity
128	Lupinol C	-11.1867	-	-
129	6,8-Diprenylgenistein	No binding affinity	-10.1819	-

130	Deguelin	No binding affinity	-7.73943	-
131	12-Deoxo-12alpha-acetoxyelliptone	No binding affinity	-7.31342	-
132	Dalbergioidin	-9.84436	-	-
133	Sinensin	No binding affinity	No binding affinity	No binding affinity
134	Neorauflavane	No binding affinity	-8.79189	-
135	Furowanin A	No binding affinity	-8.77065	-
136	Osajin	No binding affinity	-11.248	-
137	Warangalone	No binding affinity	-10.1865	-
138	Cedeodarin	No binding affinity	-9.06321	-
139	Persicoside	No binding affinity	-7.38267	-
140	Dehydrodeguelin	No binding affinity	-8.84793	-
141	8-Prenylluteone	No binding affinity	No binding affinity	No binding affinity
142	Isochandalone	No binding affinity	-12.0199	-
143	Sanggenol P	No binding affinity	No binding affinity	No binding affinity
144	3'-Geranyl-3-prenyl-2',4',5,7-tetrahydroxyflavone	No binding affinity	-9.10701	-
145	Homoferreirin	No binding affinity	-8.6318	-
146	Gancaonin M	-11.0327	-	-
147	Cedrin	No binding affinity	-8.1539	-
148	4',7-Dihydroxyflavone	-11.1563	-	-
149	Eriosemation	-12.7852	-	-
150	(2R)-8-Methylsocotrin-4'-ol	No binding affinity	-8.65627	-
151	Sappanol	-10.7911	-	-
152	Dihydrobonducellin	-10.4585	-	-
153	Eriodictyol-7-O-glucoside	No binding affinity	-8.27354	-

154	Chamaechromone	No binding affinity	No binding affinity	No binding affinity
155	Isosakuranin	No binding affinity	-9.22388	-
156	Ombuoside	No binding affinity	No binding affinity	-7.65996
157	Yukovanol	No binding affinity	-8.98169	-
158	Isoderrone	No binding affinity	No binding affinity	No binding affinity
159	Sanggenol L	No binding affinity	-10.2415	-
160	Izalpinine	-10.4285	-	-
161	Galangin 3-methyl ether	-9.75364	-	-
162	Broussoflavonol B	No binding affinity	No binding affinity	No binding affinity
163	Cathayanon I	No binding affinity	-9.76554	-
164	3'-Deoxy-4-O-methylepisappanol	-9.85585	-	-
165	3'-Deoxy-4-O-methylsappanol	-11.2685	-	-
166	4-O-Methylepisappanol	-9.4838	-	-
167	7-Hydroxyflavone	-11.6406	-	-
168	Tilianin	No binding affinity	-8.94649	-
169	6-Methoxyluteolin	No binding affinity	-8.17131	-
170	Eupatoletin	No binding affinity	-8.9384	-
171	Sanggenol A	No binding affinity	-11.549	-
172	Neocyclomorusin	No binding affinity	-9.1105	-
173	Dracoflavan B2	No binding affinity	No binding affinity	No binding affinity
174	Dracoflavan B1	No binding affinity	No binding affinity	No binding affinity
175	Dracoflavan A	No binding affinity	No binding affinity	No binding affinity
176	Dracoflavan C1	No binding affinity	No binding affinity	-8.62346
177	Dracoflavan C2	No binding affinity	No binding affinity	-8.52617
178	7,4'-Dihydroxyhomoisoflavanone	-10.8582	-	-

179	2H-1-Benzopyran-5-ol	-8.76125	-	-
180	2H-1-Benzopyran-7-yloxy	-11.9504	-	-
181	7-Hydroxy-3-(4-hydroxybenzyl)chroman	No binding affinity	No binding affinity	No binding affinity
182	Euchrestaflavanone A	No binding affinity	-10.0168	-
183	Euchrenone B1	No binding affinity	No binding affinity	No binding affinity
184	Euchrenone A10	No binding affinity	No binding affinity	No binding affinity
185	3',5'-Diprenylgenistein	No binding affinity	-9.40004	-
186	Broussoflavonol F	No binding affinity	No binding affinity	No binding affinity
187	Cathayanon H	No binding affinity	-11.9357	-
188	5,7-Dimethoxyflavanone	-10.3109	-	-
189	Sophoraflavanone C	No binding affinity	-11.0588	-
190	Pinocembrin	-11.3597	-	-
191	Dihydrooroxylin A	-10.1626	-	-
192	4'-Hydroxy-7-methoxyflavan	-10.4695	-	-
193	6-Hydroxykaempferol 3-Rutinoside -6-glucoside	No binding affinity	No binding affinity	No binding affinity
194	Eriosematin A	-10.7319	-	-
195	2''-O-Rhamnosylicariside II	No binding affinity	No binding affinity	No binding affinity
196	Icariside I	No binding affinity	No binding affinity	No binding affinity
197	Kushenol K	No binding affinity	-10.2264	-
198	Kurarinol	No binding affinity	-10.2964	-
199	Cudraflavanone B	-11.6931	-	-
200	6-Hydroxykaempferol-3,6,7-triglucoside	No binding affinity	No binding affinity	No binding affinity
201	5,6,7,4'-Tetrahydroxyflavanone 6,7-diglucoside	No binding affinity	No binding affinity	No binding affinity
202	Kaempferol 5-methyl ether	No binding affinity	No binding affinity	No binding affinity
203	Quercetin-3-o-rutinose	No binding affinity	No binding affinity	No binding affinity
204	Isothymonin	No binding affinity	No binding affinity	No binding affinity
205	Kazinol A	No binding affinity	-10.7642	-
206	5,7-Di-O-methylquercetin	No binding affinity	No binding affinity	No binding affinity

207	Onysilin	-9.54094	-	-
208	Cyclocommunol	No binding affinity	-8.93817	-
209	Cyclomulberrin	No binding affinity	-8.6714	-
210	Sanggenone K	No binding affinity	-8.5773	-
211	Sigmoidin A	No binding affinity	-8.25859	-
212	(-)-Dihydroquercetin	-9.7487	-	-
213	Kushenol C	No binding affinity	No binding affinity	No binding affinity
214	Kushenol X	-11.8145	-	-
215	7,3'-Dihydroxy-5'-methoxyisoflavone	-11.1322	-	-
216	Kushenol E	No binding affinity	-11.9239	-
217	Kaempferol-3-O-(2',6'-di-O-trans-p-coumaroyl)-beta-D-glucopyranoside	No binding affinity	No binding affinity	No binding affinity
218	Limocitrin	No binding affinity	No binding affinity	No binding affinity
219	Kaempferol 3-gentiobioside	No binding affinity	No binding affinity	No binding affinity
220	Kaempferol 3-O-(6''-galloyl)-beta-D-glucopyranoside	No binding affinity	No binding affinity	No binding affinity
221	8-Methoxykaempferol	No binding affinity	No binding affinity	No binding affinity
222	Kaempferol 3-O-beta-sophoroside	No binding affinity	No binding affinity	No binding affinity
223	6-Hydroxykaempferol 3,6-diglucoside	No binding affinity	No binding affinity	No binding affinity
224	6-Hydroxykaempferol	No binding affinity	No binding affinity	No binding affinity
225	5,7-Dihydroxychromone 7-rutinoside	No binding affinity	No binding affinity	No binding affinity
226	Didymin	No binding affinity	No binding affinity	-8.70212
227	Baicalein 6-O-glucoside	No binding affinity	-7.16036	-
228	Chrysin 6-C-glucoside	No binding affinity	-8.75617	-
229	Kushenol B	No binding affinity	-12.5758	-
230	Afrormosine	-9.0332	-	-
231	8-Prenylkaempferol	No binding affinity	No binding affinity	No binding affinity

232	Kushenol M	No binding affinity	-11.7644	-
233	Kushenol L	No binding affinity	-11.7465	-
234	Leachianone G	-11.447	-	-
235	Kushenol W	No binding affinity	-9.6031	-
236	beta-Rhamnocitrin	No binding affinity	No binding affinity	No binding affinity
237	Albanin A	No binding affinity	No binding affinity	No binding affinity
238	5,7,2',4'-Tetrahydroxy-3-geranylflavone	No binding affinity	No binding affinity	No binding affinity
239	5'-Geranyl-5,7,2',4'-tetrahydroxyflavone	No binding affinity	No binding affinity	No binding affinity
240	Kuwanon E	No binding affinity	-10.5829	-
241	Tephrosin	No binding affinity	-7.73528	-
242	Neoeriocitrin	No binding affinity	No binding affinity	No binding affinity
243	Licoflavone C	No binding affinity	No binding affinity	No binding affinity
244	Chrysin 6-C-glucoside 8-C-arabinoside	No binding affinity	No binding affinity	No binding affinity
245	Chrysin 6-C-arabinoside 8-C-glucoside	No binding affinity	No binding affinity	No binding affinity
246	Baicalin methyl ester	No binding affinity	No binding affinity	No binding affinity
247	Oroxylin A 7-O-beta-D-glucuronide methyl ester	No binding affinity	No binding affinity	No binding affinity
248	Beta-D-glucopyranosiduronic acid	No binding affinity	No binding affinity	No binding affinity
249	Glychionide A	No binding affinity	No binding affinity	-9.3146
250	Chrysin 7-O-beta-D-glucopyranuronoside	No binding affinity	No binding affinity	No binding affinity
251	Kaempferol-3-O-(6"-O-cis-coumaryl)glucoside	No binding affinity	No binding affinity	No binding affinity
252	4'-O-Methylvitexin	No binding affinity	No binding affinity	No binding affinity
253	Erythrinin A	No binding affinity	-9.26812	-
254	Corylifol C	-12.4624	-	-
255	Corylifol A	-14.5206	-	-
256	Isoneobavaisoflavone	No binding affinity	-9.79311	-
257	Neobavaisoflavone	-12.6468	-	-

258	Norwogonin	-11.3308	-	-
259	Rivularin	No binding affinity	No binding affinity	No binding affinity
260	Skullcapflavone II	No binding affinity	No binding affinity	No binding affinity
261	5,2',6'-Trihydroxy-6,7,8-trimethoxyflavone	No binding affinity	No binding affinity	No binding affinity
262	Viscidulin II	No binding affinity	No binding affinity	No binding affinity
263	Chiirirhamnin	No binding affinity	No binding affinity	No binding affinity
264	Cyanidin-3-O-sambubioside chloride	No binding affinity	No binding affinity	No binding affinity
265	Quercetin-3-O-glucuronide	No binding affinity	-7.6208	-
266	Delphinidin-3-sambubioside chloride	No binding affinity	No binding affinity	No binding affinity
267	Pelargonidin-3,5-O-diglucoside chloride	No binding affinity	No binding affinity	No binding affinity
268	7-Methoxyneochamaejasmine A	No binding affinity	No binding affinity	No binding affinity
269	Sikokianin A	No binding affinity	No binding affinity	-8.48134
270	Chamaejasmine	No binding affinity	No binding affinity	-9.22361
271	Isoneochamaejasmine A	No binding affinity	No binding affinity	-8.85314
272	Neochamaejasmine B	No binding affinity	No binding affinity	-7.72523
273	Neochamaejasmine A	No binding affinity	No binding affinity	No binding affinity
274	Isochamaejasmine	No binding affinity	No binding affinity	-8.22534
275	8-Demethylsideroxylin	No binding affinity	-8.58409	-
276	Cyanidin-3,5-O-diglucoside chloride	No binding affinity	No binding affinity	No binding affinity
277	Delphinidin-3,5-O-diglucoside chloride	No binding affinity	No binding affinity	No binding affinity
278	Delphinidin-3-O-rutinoside chloride	No binding affinity	No binding affinity	No binding affinity
279	Cyanidin-3-O-rutinoside chloride	No binding affinity	No binding affinity	-7.17165
280	Pelargonidin-3-O-glucoside chloride	No binding affinity	No binding affinity	No binding affinity
281	Pelargonidin-3-O-rutinosde chloride	No binding affinity	No binding affinity	No binding affinity
282	Pelargonidin chloride	-10.2428	-	-
283	Quercetin 3-O-[2-O-(6-O-E-feruloyl)-beta-D-glucopyranosyl]-beta-D-galactopyranoside	No binding affinity	No binding affinity	No binding affinity

284	(-)-Epigallocatechin-3-(3"-O-methyl) gallate	No binding affinity	No binding affinity	No binding affinity
285	(-)-Epicatechin-3-(3"-O-methyl) gallate	No binding affinity	No binding affinity	No binding affinity
286	Kaempferol-3-O-galactoside	No binding affinity	-8.65047	-
287	Sagittatoside C	No binding affinity	No binding affinity	No binding affinity
288	Vitexin -4"-O-glucoside	No binding affinity	No binding affinity	-7.69151
289	Neoschaftoside	No binding affinity	-7.19723	-
290	Chamaejasmenin D	No binding affinity	No binding affinity	No binding affinity
291	Isochamaejasmenin B	No binding affinity	No binding affinity	-8.81973
292	Chamaejasmenin A	No binding affinity	No binding affinity	-9.60305
293	Chamaejasmenin C	No binding affinity	No binding affinity	No binding affinity
294	Malvidin-3-O-arabinoside chloride	No binding affinity	No binding affinity	No binding affinity
295	Malvidin-3-O-glucoside chloride	No binding affinity	-8.46528	-
296	Malvidin-3-O-galactoside chloride	No binding affinity	No binding affinity	No binding affinity
297	Peonidin-3-O-arabinoside chloride	No binding affinity	-8.71413	-
298	Peonidin-3-O-glucoside chloride	No binding affinity	No binding affinity	No binding affinity
299	Peonidin-3-O-galactoside chloride	No binding affinity	No binding affinity	No binding affinity
300	Petunidin-3-O-arabinoside chloride	No binding affinity	-8.35868	-
301	Petunidin-3-O-glucoside chloride	No binding affinity	-8.32345	-
302	Petunidin-3-O-galactoside chloride	No binding affinity	-8.42737	-
303	Cyanidin-3-O-arabinoside chloride	No binding affinity	-9.19144	-
304	Cyanidin-3-O-galactoside chloride	No binding affinity	-8.4191	-
305	Delphinidin-3-O-arabinoside chloride	No binding affinity	No binding affinity	No binding affinity
306	Delphinidin-3-O-glucoside chloride	No binding affinity	No binding affinity	No binding affinity
307	Delphinidin-3-O-galactoside chloride	No binding affinity	No binding affinity	No binding affinity
308	Petunidin chloride	-9.32997	-	-

309	Malvidin chloride	No binding affinity	No binding affinity	No binding affinity
310	Peonidin chloride	No binding affinity	-7.94061	-
311	Delphinidin chloride	-9.0159	-	-
312	Vicenin -3	No binding affinity	No binding affinity	No binding affinity
313	Vicenin -2	No binding affinity	No binding affinity	-5.62812
314	Vicenin -1	No binding affinity	No binding affinity	-6.46594
315	Isoschaftoside	No binding affinity	-6.90432	-
316	6-Geranylnaringenin	No binding affinity	-12.1775	-
317	6,8-Diprenylnaringenin	No binding affinity	No binding affinity	No binding affinity
318	6-Prenylnaringenin	No binding affinity	-11.0576	-
319	8-Prenylnaringenin	No binding affinity	-9.17299	-
320	Sappanone A	-10.4363	-	-
321	Hinokiflavone	No binding affinity	No binding affinity	No binding affinity
322	Sophoraflavanone G	No binding affinity	-10.9069	-
323	2'-Methoxykurarinone	No binding affinity	-10.6296	-
324	Kurarinone	No binding affinity	-10.8024	-
325	Kushenol N	No binding affinity	No binding affinity	No binding affinity
326	Kushenol I	No binding affinity	No binding affinity	No binding affinity
327	Kushenol A	No binding affinity	-11.7606	-
328	Dracorhodin perchlorate	-11.5573	-	-
329	Cyanidin 3-sophoroside chloride	No binding affinity	No binding affinity	No binding affinity
330	Syringetin-3-O-glucoside	No binding affinity	-7.41854	-
331	Barpisoflavone A	-9.88719	-	-
332	Andrographidine C	No binding affinity	-9.1742	-

333	Andrographidine E	No binding affinity	-9.42675	-
334	Honyucitrin	No binding affinity	-9.19091	-
335	Sulfuretin	-10.6265	-	-
336	Kazinol U	-11.5505	-	-
337	5,7,4'-Trihydroxy-3,6-dimethoxy-3',5'-diprenylflavone	No binding affinity	No binding affinity	No binding affinity
338	5,7,3'-Trihydroxy-4'-methoxy-8-prenylflavanone	No binding affinity	-9.80254	-
339	Poncirin	No binding affinity	No binding affinity	No binding affinity
340	Nepetin-7-glucoside	No binding affinity	-9.01902	-
341	Centaureidin	No binding affinity	-7.96189	-
342	4-Hydroxycoumarin	-9.53165	-	-
343	Cirsilineol	No binding affinity	-7.96635	-
344	8-Prenyldaidzein	-12.0519	-	-
345	Myricetin 3-O-galactoside	No binding affinity	-4.88264	-
346	Jaceosidin	No binding affinity	-8.34264	-
347	7,3'-Dihydroxy-4'-methoxyflavan	-11.4209	-	-
348	Eurycarpin A	No binding affinity	-10.9268	-
349	Biorobin	No binding affinity	No binding affinity	-7.42637
350	2'',4''-Di-O-(Z-p-coumaroyl)afzelin	No binding affinity	No binding affinity	No binding affinity
351	7,3',4'-Trihydroxyflavone	No binding affinity	No binding affinity	No binding affinity
352	Kaempferol-3-beta-O-glucuronide	No binding affinity	-9.02152	-
353	Homoplantaginin	No binding affinity	-7.82258	-
354	Helicianeoide B	No binding affinity	No binding affinity	No binding affinity
355	Helicianeoide A	No binding affinity	No binding affinity	No binding affinity
356	Farrerol	-9.62853	-	-

357	5,7-Dihydroxychromone	No binding affinity	No binding affinity	No binding affinity
358	Capillarisin	-9.32114	-	-
359	Breviscapine	No binding affinity	No binding affinity	-8.0108
360	Cycloheterophyllin	No binding affinity	No binding affinity	No binding affinity
361	Isopedicin	-8.13321	-	-
362	Butin	-10.3299	-	-
363	Rhamnocitrin	-9.47008	-	-
364	Homopterocarpin	-9.37792	-	-
365	Demethoxycapillarisin	-9.42241	-	-
366	Isosilybin	No binding affinity	-7.71053	-
367	Cyclomorusin	No binding affinity	-8.65798	-
368	6,8-Diprenylorobol	No binding affinity	No binding affinity	No binding affinity
369	5,8,4'-Trihydroxy-7-methoxyflavone 8-O-glucoside	No binding affinity	No binding affinity	No binding affinity
370	Genistin	No binding affinity	-9.42381	-
371	Silychristin	No binding affinity	-8.79219	-
372	Silydianin	No binding affinity	-7.5102	-
373	Tangeretin	-9.32346	-	-
374	Derrone	No binding affinity	-10.395	-
375	2'-Hydroxydaidzein	-10.3022	-	-
376	3'-Hydroxy Puerarin	No binding affinity	No binding affinity	No binding affinity
377	6-Methoxykaempferol 3-O-rutinoside	No binding affinity	No binding affinity	No binding affinity
378	5-Hydroxy-7,8,2',5'-tetramethoxyflavone 5-O-glucoside	No binding affinity	No binding affinity	No binding affinity
379	Isolicoflavonol	No binding affinity	No binding affinity	No binding affinity
380	Hydroxytuberosone	No binding affinity	-8.57378	-
381	8-Isomulberrin hydrate	No binding affinity	No binding affinity	No binding affinity
382	Kaempferol 3-O-arabinoside	No binding affinity	-8.73101	-

383	Kazinol B	No binding affinity	-10.0561	-
384	Ligustroflavone	No binding affinity	No binding affinity	No binding affinity
385	Isothymusin	No binding affinity	-8.5445	-
386	Leachianone A	No binding affinity	-10.473	-
387	3,4'-Dihydroxy-3,5',7-trimethoxyflavan	No binding affinity	No binding affinity	No binding affinity
388	Sagittatoside B	No binding affinity	No binding affinity	No binding affinity
389	(-)-Epigallocatechin(EGC)	-9.26236	-	-
390	Diosmetin	No binding affinity	-8.54945	-
391	Viscidulin III tetraacetate	-10.813	-	-
392	5,7-Dihydroxy-2-isopropylchromone	No binding affinity	No binding affinity	No binding affinity
393	Schaftoside	No binding affinity	No binding affinity	-6.54653
394	5-Hydroxy-7-acetoxy-8-methoxyflavone	No binding affinity	No binding affinity	No binding affinity
395	Oroxylin A 7-O-beta-D-glucuronide	No binding affinity	-10.9635	-
396	Eupatilin	No binding affinity	No binding affinity	-7.8068
397	Prunetin	No binding affinity	No binding affinity	No binding affinity
398	Artemetin acetate	No binding affinity	No binding affinity	No binding affinity
399	6-Methoxynaringenin	No binding affinity	No binding affinity	-8.31833
400	Isoginkgetin	No binding affinity	No binding affinity	No binding affinity
401	Ginkgetin	No binding affinity	No binding affinity	No binding affinity
402	Theaflavin-3-gallate	No binding affinity	No binding affinity	No binding affinity
403	Apiin	No binding affinity	-9.52605	-
404	DL-Catechin	-10.342	-	-
405	8-O-Demethyl-7-O-methyl-3,9-dihydropunctatin	No binding affinity	No binding affinity	No binding affinity
406	Viscidulin I	No binding affinity	-10.2253	-
407	Viscidulin III	No binding affinity	No binding affinity	-8.52174
408	8-Methoxybonducellin	No binding affinity	-8.37828	-

409	Kaempferol-7-O-D-glucopyranoside	No binding affinity	-7.52383	-
410	Sophoricoside	No binding affinity	No binding affinity	-7.43717
411	Sagittatoside A	No binding affinity	No binding affinity	No binding affinity
412	Ikarisoside F	No binding affinity	No binding affinity	No binding affinity
413	prim-O-Glucosylangelicain	No binding affinity	-7.93295	-
414	Eupatorin	No binding affinity	-7.91621	-
415	Heteronoside	No binding affinity	No binding affinity	No binding affinity
416	Anisofolin A	No binding affinity	No binding affinity	No binding affinity
417	4',5-Dihydroxy-3',5',6,7-tetramethoxyflavone	-8.54233	-	-
418	Visnagin	-8.13139	-	-
419	Khellin	-7.26813	-	-
420	2',5,6',7-Tetraacetoxyflavanone	No binding affinity	-8.99237	-
421	2',5,6',7-Tetrahydroxyflavanone	No binding affinity	No binding affinity	No binding affinity
422	Padmatin	-9.79792	-	-
423	2',3,5,6',7-Pentahydroxyflavanone	No binding affinity	No binding affinity	No binding affinity
424	Blumeatin B	-9.25608	-	-
425	3-Hydroxy-4',5,7-trimethoxyflavanone	No binding affinity	No binding affinity	No binding affinity
426	Lupalbigenin	No binding affinity	No binding affinity	No binding affinity
427	5-Deoxycajanin	-9.63669	-	-
428	Artocarpin	No binding affinity	-8.29308	-
429	Isomedicarpin	-9.69976	-	-
430	4',4'''-Di-O-methylcupressuflavone	No binding affinity	No binding affinity	No binding affinity
431	2,3-Dehydrokievitone	No binding affinity	-10.7816	-
432	Sepinol	-9.58256	-	-
433	Dihydrotamarixetin	-9.33093	-	-
434	6-Acetyl-2,2-dimethylchroman-4-one	-9.68639	-	-

435	Isowighteone	-12.3464	-	-
436	4',5,7-Trihydroxy-6-prenylflavone	No binding affinity	No binding affinity	No binding affinity
437	Trifolirhizin	No binding affinity	-7.61657	-
438	2,3,2",3"-Tetrahydroochnaflavone	No binding affinity	No binding affinity	No binding affinity
439	Hydrangenol 8-O-glucoside	No binding affinity	-9.37534	-
440	5-Hydroxy-7-acetoxyflavone	-11.5705	-	-
441	5,7-Diacetoxyflavone	-11.242	-	-
442	4',5-Dihydroxyflavone	No binding affinity	No binding affinity	No binding affinity
443	Kaempferol 3-O-(6"-O-acetyl)glucoside-7-O-rhamnoside	No binding affinity	No binding affinity	-6.92692
444	Cirsimaritin	No binding affinity	-8.17873	-
445	alpha-Isowighteone	No binding affinity	No binding affinity	No binding affinity
446	Dihydroalpinumisoflavone	No binding affinity	-8.76075	-
447	Erythrinin C	No binding affinity	-8.07209	-
448	Wogonin	No binding affinity	No binding affinity	No binding affinity
449	Morusinol	No binding affinity	-8.42978	-
450	Mulberrin	No binding affinity	-10.9287	-
451	Morusin	No binding affinity	-9.44902	-
452	Saponarin	No binding affinity	No binding affinity	No binding affinity
453	Isosaponarin	No binding affinity	No binding affinity	No binding affinity
454	Meloside A	No binding affinity	No binding affinity	-8.86245
455	Vaccarin	No binding affinity	No binding affinity	No binding affinity
456	5-O-Methylnaringenin	-8.47876	-	-
457	3,9-Dihydroxypterocarpan	No binding affinity	No binding affinity	No binding affinity
458	Isobonducellin	-10.704	-	-
459	Tamarixetin	-9.36103	-	-
460	Chrysosplenetin	No binding affinity	-6.92771	-

461	Isosativan	-9.96454	-	-
462	Sissotrin	No binding affinity	-8.87035	-
463	Cosmosiin	No binding affinity	-8.91168	-
464	Carpachromene	No binding affinity	-9.62374	-
465	Engeletin	No binding affinity	No binding affinity	No binding affinity
466	Avicularin	No binding affinity	-9.10002	-
467	4'-Hydroxywogonin	No binding affinity	No binding affinity	No binding affinity
468	Penduletin	No binding affinity	No binding affinity	-8.29689
469	8-Demethyleucalyptin	No binding affinity	-8.61513	-
470	Steppogenin	No binding affinity	-8.9348	-
471	Isorhoifolin	No binding affinity	No binding affinity	-7.41496
472	Galangin	-10.4538	-	-
473	Pinobanksin	-10.4823	-	-
474	5-Hydroxy-7,8-dimethoxyflavanone	-10.5051	-	-
475	Isoastilbin	No binding affinity	-7.89381	-
476	Corylin	No binding affinity	-9.3426	-
477	Flavaprin	No binding affinity	No binding affinity	-8.12226
478	Apigenin 7-O-methylglucuronide	No binding affinity	No binding affinity	No binding affinity
479	Prunin	No binding affinity	-8.58407	-
480	Myricetin	No binding affinity	-8.49072	-
481	Ombuin	-9.12871	-	-
482	Scillascillin	No binding affinity	-8.11249	-
483	Phellamurin	No binding affinity	-9.71499	-
484	Quercetin 3-O-robinobioside	No binding affinity	No binding affinity	-9.2417

485	Quercitrin	No binding affinity	-7.95071	-
486	Sciadopitysin	No binding affinity	No binding affinity	No binding affinity
487	Bilobetin	No binding affinity	No binding affinity	No binding affinity
488	3-O-Acetylpinobanksin	-10.5032	-	-
489	2-Hydroxy-7-O-methylscillascillin	No binding affinity	-7.3412	-
490	Apigenin	No binding affinity	-9.36897	-
491	Hesperetin	-10.3912	-	-
492	Tricin	No binding affinity	No binding affinity	No binding affinity
493	Tectochrysin	-11.1925	-	-
494	Hesperidin	No binding affinity	No binding affinity	-8.8848
495	Kaempferol	-10.0098	-	-
496	7-O-Methyleriodictyol	-9.99711	-	-
497	7,4'-Di-O-methylapigenin	No binding affinity	-8.39579	-
498	5,7-Diacetoxy-3,4',8-trimethoxyflavone	No binding affinity	No binding affinity	No binding affinity
499	Wighteone	No binding affinity	-9.83925	-
500	Taiwanhomoflavone B	No binding affinity	No binding affinity	No binding affinity
501	Theaflavin-3'-gallate	No binding affinity	No binding affinity	No binding affinity
502	Theaflavine-3,3'-digallate	No binding affinity	No binding affinity	No binding affinity
503	Theaflavin	No binding affinity	No binding affinity	-8.0354
504	Methylophiopogonanone B	-11.9125	-	-
505	Methylophiopogonanone A	-11.0066	-	-
506	Araneosol	No binding affinity	No binding affinity	No binding affinity
507	Angelicain	-8.92644	-	-
508	Chrysoeriol	No binding affinity	No binding affinity	No binding affinity
509	Luteolin	-10.1259	-	-
510	Baicalein	-10.9568	-	-
511	Kaempferide	-9.70306	-	-

512	Epicatechin	-10.342	-	-
513	Robinetin	-8.93622	-	-
514	Daidzein	-10.3432	-	-
515	Afzelin	No binding affinity	-9.02415	-
516	Kaempferitrin	No binding affinity	No binding affinity	No binding affinity
517	Tetrahydroamentoflavone	No binding affinity	No binding affinity	No binding affinity
518	Hyperoside	No binding affinity	No binding affinity	No binding affinity
519	Isoquercitrin	No binding affinity	-7.47242	-
520	Hydrangenol	-11.1564	-	-
521	Acacetin	No binding affinity	-9.2237	-
522	Isosakuranetin	-11.1911	-	-
523	Naringenin	-10.6376	-	-
524	Chrysin	-11.3443	-	-
525	Pinocembrin	-11.3637	-	-
526	Pinostrobin	-11.0596	-	-
527	Linarin	No binding affinity	No binding affinity	-6.49803
528	Orobol	-9.51625	-	-
529	Aromadendrin	-10.3669	-	-
530	Isorhamnetin	-8.60312	-	-
531	Quercetin	-9.6088	-	-
532	Astragalin	No binding affinity	-8.72426	-
533	Artemetin	No binding affinity	-7.2743	-
534	Nobiletin	No binding affinity	-7.18921	-
535	Brazilin	No binding affinity	-8.82891	-
536	Genistein	No binding affinity	-9.48141	-
537	Norkhellol	-8.47776	-	-

538	Robtin	-10.0583	-	-
539	Dihydrorobinetin	-10.0583	-	-
540	Genkwanin	-10.3809	-	-
541	Catechin 7-xyloside	No binding affinity	-8.1075	-
542	Glabranin	-13.2895	-	-
543	Luteone	No binding affinity	-9.67007	-
544	Anhydrotuberosin	No binding affinity	-9.97014	-
545	Skullcapflavone I	No binding affinity	No binding affinity	No binding affinity
546	Taxifolin 3-O-beta-D-xylopyranoside	No binding affinity	No binding affinity	No binding affinity
547	Luteolin 7,3'-di-O-glucuronide	No binding affinity	No binding affinity	-7.63552
548	Luteollin 5-glucoside	No binding affinity	No binding affinity	No binding affinity
549	Luteolin-7-O-glucoside	No binding affinity	-7.72464	-
550	Isovitexin	No binding affinity	-6.76593	-
551	3,4,4',7-Tetrahydroxyflavan	-11.3652	-	-
552	Scutellarein	No binding affinity	-8.24088	-
553	Naringenin trimethyl ether	No binding affinity	-7.87226	-
554	Cimifugin	-8.89443	-	-
555	Phaseollidin	-10.3904	-	-
556	7,4'-Dihydroxy-3'-prenylflavan	-13.2891	-	-
557	Naringenin triacetate	-10.7046	-	-
558	Isohemiphloin	No binding affinity	-10.1394	-
559	Vitexin	No binding affinity	-9.56847	-
560	3'-O-Methylorobol	No binding affinity	No binding affinity	No binding affinity
561	Oroxylin A	-10.3017	-	-
562	Alpinetin	-10.7255	-	-
563	Vestitol	-10.7681	-	-

564	Moslosooflavone	No binding affinity	No binding affinity	No binding affinity
565	Noricaritin	No binding affinity	No binding affinity	No binding affinity
566	Icaritin	No binding affinity	No binding affinity	No binding affinity
567	β-Anhydroicaritin	No binding affinity	No binding affinity	No binding affinity
568	Baohuoside I	No binding affinity	-9.80254	-
569	Maohuoside A	No binding affinity	No binding affinity	No binding affinity
570	4'-Demethyleucomin	No binding affinity	-9.85862	-
571	Luteolin-7-O-glucuronide	No binding affinity	-7.81076	-
572	Prudomestin	No binding affinity	No binding affinity	No binding affinity
573	Isoapetalic acid	-12.0376	-	-
574	Alpinumisoflavone	-9.91228	-	-
575	(-)-Catechin gallate(CG)	No binding affinity	-8.69806	-
576	Diosmetin-7-O-beta-D-glucopyranoside	No binding affinity	No binding affinity	No binding affinity
577	Pachypodol	-9.02605	-	-
578	Quercetin 3,4'-dimethyl ether	-8.89235	-	-
579	Kumatakenin	-9.97667	-	-
580	Cajanin	-9.57218	-	-
581	Kaempferol 3-neohesperidoside	No binding affinity	No binding affinity	No binding affinity
582	Apigenin-7-glucuronide	No binding affinity	-8.73298	-
583	Medicarpin	-10.1432	-	-
584	Hesperetin 7-O-glucoside	No binding affinity	No binding affinity	No binding affinity
585	5,7-Dihydroxy-6,8-dimethoxyflavone	No binding affinity	No binding affinity	No binding affinity
586	Isobavachin	-13.7368	-	-
587	Eucalyptin	No binding affinity	-8.75817	-
588	Sideroxylin	No binding affinity	No binding affinity	No binding affinity
589	5,7,3'-Trihydroxy-6,4',5'-trimethoxyflavanone	No binding affinity	No binding affinity	No binding affinity
590	Mearnsitrin	No binding affinity	-7.49177	-

591	Robinin	No binding affinity	No binding affinity	No binding affinity
592	Vitexin-2"-O-rhamnoside	No binding affinity	No binding affinity	-8.89106
593	Fisetin	No binding affinity	No binding affinity	No binding affinity
594	Astilbin	No binding affinity	-8.74676	-
595	Sakuranetin	-10.1739	-	-
596	4',7-Di-O-methylnaringenin	-9.97816	-	-
597	5-Hydroxy-3',4',7-trimethoxyflavone	No binding affinity	No binding affinity	No binding affinity
598	Vitexicarpin	No binding affinity	-7.39589	-
599	Apigenin 5-O-beta-D-glucopyranoside	No binding affinity	-8.33244	-
600	Leucoside	No binding affinity	No binding affinity	No binding affinity
601	Ampelopsin	-9.29579	-	-
602	Typhaneoside	No binding affinity	No binding affinity	No binding affinity
603	Diosimin	No binding affinity	No binding affinity	-8.09267
604	Luteolin-6-C-glucoside	No binding affinity	No binding affinity	-7.5404
605	Orientin	No binding affinity	No binding affinity	No binding affinity
606	5-Hydroxy-7-methoxy-3-(4-hydroxybenzylidene)chroman-4-one	No binding affinity	No binding affinity	No binding affinity
607	Velutin	No binding affinity	-8.04894	-
608	(+)-Afzelechin	-10.5911	-	-
609	(-)-Epiafzelechin	-10.7312	-	-
610	Putraflavone	No binding affinity	No binding affinity	No binding affinity
611	5,7-Diacetoxy-8-methoxyflavone	-11.4382	-	-
612	Heveaflavone	No binding affinity	No binding affinity	No binding affinity
613	Desmethoxycentaureidin	No binding affinity	-8.2049	-
614	Amaronol B	-8.34322	No binding affinity	-
615	Amaronol A	-9.94236	No binding affinity	-
616	Guaijaverin	No binding affinity	-8.02485	-
617	Podocarpusflavone A	No binding affinity	No binding affinity	No binding affinity

618	Lethedioside A	No binding affinity	No binding affinity	-7.62995
619	Lethedoside A	No binding affinity	-8.58434	-
620	7,4'-Di-O-methylapigenin 5-O-xylosylglucoside	No binding affinity	No binding affinity	No binding affinity
621	Erysubin B	No binding affinity	-9.45089	-
622	Erysubin A	No binding affinity	-10.2175	-
623	Agrimonolide	-11.1908	-	-
624	Barbacarpan	No binding affinity	-8.05471	-
625	2',4',5'-Trimethoxy-2'',2''-dimethylpyrano[5'',6'':6,7]isoflavone	No binding affinity	No binding affinity	No binding affinity
626	Ermanin	-9.56284	-	-
627	Formononetin	-10.8312	-	-
628	Fustin	-9.82291	-	-
629	(-)-Maackiain	-9.20281	-	-
630	Tiliroside	No binding affinity	No binding affinity	-9.19077
631	8-Hydroxy-3,5,7,3',4',5'-hexamethoxyflavone	No binding affinity	No binding affinity	No binding affinity
632	Hydroxygenkwanin	No binding affinity	-8.57192	-
633	Epicatechin pentaacetate	No binding affinity	-9.59836	-
634	Bavachin	No binding affinity	-11.4624	-
635	Bavachinin	No binding affinity	-11.4442	-
636	Epimedin C	No binding affinity	No binding affinity	No binding affinity
637	7,4-Di-O-methylapigenin 5-O-glucoside	No binding affinity	No binding affinity	No binding affinity
638	Epimedin B	No binding affinity	No binding affinity	No binding affinity
639	Epimedin A	No binding affinity	No binding affinity	No binding affinity
640	Salvigenin	No binding affinity	No binding affinity	No binding affinity
641	Morin	-10.34	-	-
642	Tectoridin	No binding affinity	-8.95672	-
643	Tectorigenin	-10.184	-	-

644	Dihydromorin	No binding affinity	No binding affinity	No binding affinity
645	Naringenin-4',7-diacetate	No binding affinity	-8.67929	-
646	Myricitrin	No binding affinity	-7.54049	-
647	Nicotiflorin	No binding affinity	No binding affinity	-7.56707
648	Irisflorentin	No binding affinity	-6.98046	-
649	Lysionotin	No binding affinity	No binding affinity	No binding affinity
650	Herbacetin	-9.99926	-	-
651	Ficusin A	No binding affinity	-9.55358	-
652	Rhoifolin	No binding affinity	No binding affinity	-6.84704
653	3',4',5',3,5,6,7-Heptamethoxyflavone	No binding affinity	-6.03464	-
654	Narcissoside	No binding affinity	No binding affinity	-8.09236
655	Grosvenorin	No binding affinity	No binding affinity	No binding affinity
656	Quercetin-7-O-beta-D-glucopyranoside	No binding affinity	No binding affinity	No binding affinity
657	Kaempferol tetraacetate	No binding affinity	-9.23867	-
658	3-Hydroxy-5,7-dimethoxy-3',4'-methylenedioxyflavan	No binding affinity	No binding affinity	No binding affinity
659	Catechin pentaacetate	No binding affinity	-9.61828	-
660	Rhodionin	No binding affinity	-10.0709	-
661	Isorhamnetin-3-O-beta-D-Glucoside	No binding affinity	-7.83604	-
662	Maackiain	-10.1982	-	-
663	3',5,5',7-Tetrahydroxyflavanone	-9.89205	-	-
664	Isorhamnetin-3-O-neohespeidoside	No binding affinity	No binding affinity	No binding affinity
665	Sec-O-Glucosylhamaudol	No binding affinity	No binding affinity	No binding affinity
666	Cyanidin Chloride	No binding affinity	-8.55676	-
667	Cyanidin-3-O-glucoside chloride	No binding affinity	-8.55676	-
668	5,7-Dihydroxy-3,4',8-trimethoxyflavone	No binding affinity	No binding affinity	-7.43044

669	Biochanin A	No binding affinity	No binding affinity	No binding affinity
670	Glabridin	No binding affinity	-9.78974	-
671	2''-O-Galloylhyperin	No binding affinity	No binding affinity	No binding affinity
672	Pectolinarin	No binding affinity	No binding affinity	-6.14422
673	3,5-Dihydroxy-4',7-dimethoxyflavone	No binding affinity	No binding affinity	No binding affinity
674	Catechin	-10.1718	-	-
675	Eriodictyol	-10.5055	-	-
676	Eriocitrin	No binding affinity	-6.94665	-
677	Rutin	No binding affinity	-6.94665	-
678	Oroxin B	No binding affinity	No binding affinity	-7.8487
679	Oroxin A	No binding affinity	-10.5728	-
680	Wogonoside	No binding affinity	-9.30845	-
681	Sanggenone D	No binding affinity	No binding affinity	No binding affinity
682	Spinosin	No binding affinity	No binding affinity	-9.57997
683	Sinensetin	No binding affinity	-8.05549	-
684	Sanggenone C	No binding affinity	No binding affinity	No binding affinity
685	Chrysosplenol D	No binding affinity	No binding affinity	No binding affinity
686	3-O-Methylquercetin	-9.99903	-	-
687	Methyl hesperidin	No binding affinity	No binding affinity	-7.80653
688	3-O-Methylquercetin tetraacetate	No binding affinity	No binding affinity	No binding affinity
689	Thunberginol C	-10.7402	-	-
690	(-)-Gallocatechin gallate	No binding affinity	-8.52086	-
691	(-)-Epicatechin gallate	No binding affinity	-9.15537	-
692	(-)-Epigallocatechin gallate	No binding affinity	No binding affinity	No binding affinity
693	Procyanidin C1	No binding affinity	No binding affinity	No binding affinity
694	Procyanidin B3	No binding affinity	No binding affinity	No binding affinity

695	Procyanidin B2	No binding affinity	No binding affinity	-9.5615
696	Procyanidin B1	No binding affinity	No binding affinity	-9.72862
697	Proanthocyanidins	No binding affinity	No binding affinity	No binding affinity
698	Naringin	-10.7042	-	-
699	Hispidulin	No binding affinity	-8.27935	-
700	Icariin	No binding affinity	No binding affinity	No binding affinity
701	Narirutin	No binding affinity	No binding affinity	-8.81093
702	Silymarin	No binding affinity	No binding affinity	-7.24005
703	Amentoflavone	No binding affinity	No binding affinity	No binding affinity
704	Complanatoside A	No binding affinity	No binding affinity	No binding affinity
705	Jaceidin triacetate	No binding affinity	-7.91887	-
706	Kaempferol 3,4,7-triacetate	No binding affinity	-9.55387	-
707	(+)-Taxifolin	-9.89	-	-
708	Glyasperin A	No binding affinity	No binding affinity	No binding affinity
709	Astragaloside	No binding affinity	No binding affinity	No binding affinity
710	Puerarin	No binding affinity	-8.07091	-
711	Eucalyptin acetate	No binding affinity	-7.89546	-
712	Liquiritigenin	-10.7165	-	-
713	Liquiritin	No binding affinity	-7.80837	-
714	Epimedin A1	No binding affinity	No binding affinity	No binding affinity
715	Calycosin-7-O-beta-D-glucoside	No binding affinity	-9.63884	-
716	Calycosin	-9.7111	-	-
717	Glucosylvitexin	No binding affinity	No binding affinity	No binding affinity
718	(+)-Gallocatechin	-9.09706	-	-
719	(-)-Gallocatechin	-9.1132	-	-
720	Ononin	No binding affinity	-8.44231	-

721	Theaflavin 3,3'-di-O-gallate	No binding affinity	No binding affinity	No binding affinity
722	Neohesperidin	No binding affinity	No binding affinity	No binding affinity
723	Scutellarin	No binding affinity	No binding affinity	-7.48917
724	Baicalin	No binding affinity	-9.07433	-
725	Exoticin	No binding affinity	-6.27277	-
726	Apigenin 4'-O-rhamnoside	No binding affinity	No binding affinity	-6.4208
727	5-Acetoxy-7-hydroxyflavone	-10.5609	-	-
728	Glycitein	-10.3463	-	-
729	Glycitin	No binding affinity	No binding affinity	-9.26076
730	Daidzin	No binding affinity	-9.43005	-
731	Taxifolin-3-glucopyranoside	No binding affinity	-8.45622	-
732	Agrimonolide 6-O-glucoside	No binding affinity	-9.01143	-
733	3',5,5',7-Tetrahydroxy-4',6-dimethoxyflavone	No binding affinity	No binding affinity	No binding affinity
734	6''-O-acetylisovitexin	No binding affinity	No binding affinity	-7.45894
735	6''-O-Acetylastragalin	No binding affinity	-8.80276	-
736	Blumeatin	-9.59687	-	-
737	Artoheterophyllin B	No binding affinity	-11.062	-
738	Quercetin	-9.60998	-	-
739	3',4',7-Trimethoxyflavan	-84668	-	-
740	2'-Hydroxygenistein	No binding affinity	-9.6421	-
741	5-Hydroxy-7,8-dimethoxyflavanone	-10.4888	-	-
742	(3S)-Hydrangenol 8-O-glucoside pentaacetate	No binding affinity	-8.57758	-
743	(3R)-Hydrangenol 8-O-glucoside pentaacetate	No binding affinity	-8.00538	-
744	3-Deoxysappanone B	-10.331	-	-
745	2',5,7-Trihydroxy-8-methoxyflavanone	No binding affinity	No binding affinity	No binding affinity
746	3',5-Dihydroxy-4',5',6,7-tetramethoxyflavone	No binding affinity	No binding affinity	No binding affinity
747	Pinocembrin diacetate	-11.7352	-	-

748	7,3',4'-Trihydroxy-3-benzyl-2H-chromene	-11.6869	-	-
749	7-Hydroxy-3-(4-hydroxybenzylidene)chroman-4-one	No binding affinity	No binding affinity	No binding affinity
750	Pinocembrin 7-acetate	-12.0458	-	-
751	4-Demethyl-3,9-dihydroeucomin	No binding affinity	No binding affinity	No binding affinity
752	3'-Hydroxy-3,9-dihydroeucomin	-10.395	-	-
753	4-O-Methylsappanol	-9.8726	-	-
754	Catechin 3-rhamnoside	No binding affinity	No binding affinity	-8.08842
755	3,7-O-Diacetylpinobanksin	No binding affinity	-9.60935	-
756	Jaceidin	No binding affinity	-8.26015	-
757	Noreugenin	-8.27185	-	-
758	3,8'-Biapigenin	No binding affinity	No binding affinity	No binding affinity
759	7-Hydroxy-2',5,8-trimethoxyflavanone	No binding affinity	-8.6775	-
760	Pectolinarigenin	No binding affinity	-8.61724	-
761	Geraldol	-8.85003	-	-
762	Gossypin	No binding affinity	No binding affinity	No binding affinity
763	Myrecitin	No binding affinity	No binding affinity	No binding affinity
764	Lanceolatin	No binding affinity	No binding affinity	No binding affinity
765	Kanjone	-11.5015	-	-
766	Hesperedin	No binding affinity	No binding affinity	No binding affinity
767	Epigallocatechin	-9.38181	-	-
768	Epicatechin Gallate	No binding affinity	-9.64346	-
769	Epigallactocatechin Gallate	No binding affinity	No binding affinity	No binding affinity
770	Theaflavins	No binding affinity	No binding affinity	-7.67352
771	2,4 - Dihydroxychalcone	No binding affinity	No binding affinity	No binding affinity
772	Isoliquiritigenin	-12.0208	-	-
773	4,4 - Dihydroxychalcone	No binding affinity	No binding affinity	-8.28091

774	2,5 - Dihydroxychalcone	-12.0206	-	-
775	2,2 - Dihydroxychalcone	-12.2516	-	-
776	Equol	-10.4969	-	-
777	6-Aminoflavone	-10.6331	-	-
778	7,8- Benzoflavone	No binding affinity	No binding affinity	No binding affinity
779	Diosmin	No binding affinity	-7.41212	-
780	Karanjin	-11.1299	-	-
781	5- Methoxyflavone	-11.6729	-	-
782	Primuletin	No binding affinity	-9.13353	-
783	Avicularian	No binding affinity	-9.18386	-
784	Vicenin-2	No binding affinity	No binding affinity	-5.89102
785	Cyanidin	No binding affinity	No binding affinity	-5.67817
786	Delphinidin	No binding affinity	No binding affinity	-5.74919
787	Malvidin	No binding affinity	No binding affinity	-5.47788
788	Pelargonidin	-10.2428	-	-
789	Peonidin	-9.14765	-	-
790	Petunidin	-9.32997	-	-
791	Anthocyanin	-13.0199	-	-
792	4 Methoxy 4 Hydroxy Chalcone	No binding affinity	No binding affinity	No binding affinity
793	Purpurin	-10.9723	-	-
794	Cisplatin	No binding affinity	No binding affinity	No binding affinity
795	Clitocine	-6.3948	-	-
796	Pongamol	-13.4919	-	-
797	Hyperin	No binding affinity	-7.52333	-
798	Flavopiridol	No binding affinity	No binding affinity	No binding affinity
799	Indirubin	-11.2671	-	-

800	Xanthone	-10.7763	-	-
801	Isoquercetin	No binding affinity	-8.14798	-
802	Resveratol	-12.6598	-	-
803	Silibinin	No binding affinity	No binding affinity	-6.25913

Supplementary Table S2: Lipinski's rule of five properties of phytochemicals used in the study having binding affinity energies above 10 kcal/mol.

S.NO.	PHYTOCHEMICALS	BINDING AFFINITY	miLog P	TPSA	natoms	MW	nON	nOHNH	nrotb	VOLUME
1	Isobavachin	-13.7368	4.45	66.76	24	324.38	4	2	3	299.58
2	Glabranin	-13.2895	4.85	66.76	24	324.38	4	2	3	299.58
3	Anthocyanin	-13.0199	1.31	11.17	16	207.25	1	0	1	194.72
4	Eriosemation	-12.7852	4.85	70.67	23	314.38	4	2	4	299.3
5	Resveratol (NF)	-12.6598	2.99	60.68	17	228.25	3	3	2	206.92
6	Neobavaisoflavone	-12.6468	4.81	70.67	24	322.36	4	2	3	293.37
7	Corylifol C	-12.4624	4.52	90.89	25	338.36	5	3	3	301.39
8	Isowighteone	-12.3464	4.52	90.89	25	338.36	5	3	3	301.39
9	2,2-Dihydroxychalcone	-12.2516	4.01	57.53	18	240.26	3	2	3	217.89
10	8-Prenyldaidzein	-12.0519	4.81	70.67	24	322.36	4	2	3	293.37
11	Pinocembrin 7-acetate	-12.0458	2.63	72.84	22	298.29	5	1	3	258.75
12	Isoapetalic acid	-12.0376	2.38	93.07	28	388.46	6	2	5	361.95
13	Isoliquiritigenin	-12.0208	2.77	77.75	19	256.26	4	3	3	225.91
14	2,5-Dihydroxychalcone	-12.0206	3.25	57.53	18	240.26	3	2	3	217.89
15	Glepidotin B	-12.0186	3.93	86.99	25	340.38	5	3	3	307.63
16	2H-1-Benzopyran-7-yloxy	-11.9504	2.68	46.53	18	240.26	3	1	1	214.23
17	Methylophiopogonanone B	-11.9125	3.78	76	24	328.36	5	2	3	297.71
18	Isoxanthohumol	-11.7745	4.44	76	26	354.4	5	2	4	325.13
19	Kushenol L	-11.7465	4.74	127.44	32	440.49	7	5	5	401
20	Pinocembrin diacetate	-11.7352	2.19	78.92	25	340.33	6	0	5	295.27

21	Cudraflavanone B	-11.6931	4.08	107.22	26	356.37	6	4	3	315.62
22	7,3’,4’-Trihydroxy-3-benzyl-2H-chromene	-11.6869	2.62	69.92	20	270.28	4	3	2	238.67
23	5-Methoxyflavone	-11.6729	3.75	39.45	19	252.27	3	0	2	225.54
24	7-Hydroxyflavone	-11.6406	3.23	50.44	18	238.24	3	1	1	208.01
25	5-Hydroxy-7-acetoxyflavone	-11.5705	2.97	76.74	22	296.28	5	1	3	252.54
26	Dracorhodin perchlorate	-11.5573	1.63	40.63	20	267.3	3	1	2	244.84
27	Kazinol U	-11.5505	4.63	69.92	24	326.39	4	3	3	305.42
28	Dihydrodaidzein	-11.5459	2.42	66.76	19	256.26	4	2	1	222.24
29	Kanjone	-11.5015	4	52.59	22	292.29	4	0	2	251.1
30	Bavachin	-11.4624	4.45	66.76	24	324.38	4	2	3	299.58
31	Leachianone G	-11.447	4.28	107.22	26	356.37	6	4	3	315.62
32	Bavachinin	-11.4442	4.52	55.77	25	338.4	4	1	4	317.11
33	5,7-Diacetoxy-8-methoxyflavone	-11.4382	2.53	92.06	27	368.34	7	0	6	314.6
34	7,3'-Dihydroxy-4'-methoxyflavan	-11.4209	2.92	58.92	20	272.3	4	2	2	245.61
35	Pinocembrin	-11.3637	2.6	66.76	19	256.26	4	2	1	222.24
36	Chrysin	-11.3443	2.94	70.67	19	254.24	4	2	1	216.03
37	Norwogonin	-11.3308	2.68	90.89	20	270.24	5	3	1	224.05
38	3'-Deoxy-4-O-methylsappanol	-11.2685	2	79.15	22	302.33	5	3	3	270.13
39	Indirubin	-11.2671	2.9	65.72	20	262.27	4	2	0	223.96
40	5,7-Diacetoxyflavone	-11.242	2.75	82.82	25	338.31	6	0	5	289.05
41	Tectochrysin	-11.1925	3.48	59.67	20	268.27	4	1	2	233.56
42	Isosakuranetin	-11.1911	2.65	76	21	286.28	5	2	2	247.79
43	Agrimonolide	-11.1908	3.54	76	23	314.34	5	2	4	281.39
44	Lupinol C	-11.1867	3.15	116.45	27	370.36	7	4	2	313.46
45	Hydrangenol	-11.1564	2.81	66.76	19	256.26	4	2	1	222.24
46	4',7-Dihydroxyflavone	-11.1563	2.75	70.67	19	254.24	4	2	1	216.03

47	7,3'-Dihydroxy-5'-methoxyisoflavone	-11.1322	2.54	79.9	21	284.27	5	2	2	241.58
48	Karanjin	-11.1299	4	52.59	22	292.29	4	0	2	251.1
49	Pinostrobin	-11.0596	3.13	55.77	20	270.28	4	1	2	239.77
50	6-Prenylnaringenin	-11.0576	4.16	86.99	25	340.38	5	3	3	307.6
51	Bidwillol A	-11.0226	4.94	58.92	25	338.4	4	2	4	316.74
52	Methylophiopogonanone A	-11.0066	3.78	76	24	328.36	5	2	3	297.71
53	Purpurin	-10.9723	2.61	94.83	19	256.21	5	3	0	206.63
54	Baicalein	-10.9568	2.68	90.89	20	270.24	5	3	1	224.05
55	Eurycarpin A	-10.9268	4.52	90.89	25	338.36	5	3	3	301.39
56	7,4'-Dihydroxyhomoisoflavanone	-10.8582	2.52	66.76	20	270.28	4	2	2	239.04
57	Formononetin	-10.8312	3.1	59.67	20	268.27	4	1	2	233.56
58	Sappanol	-10.7911	0.75	110.37	22	304.3	6	5	2	260.62
59	2,3-Dehydrokievitone	-10.7816	4.23	111.12	26	354.36	6	4	3	309.41
60	Xanthone	-10.7763	3.57	30.21	15	196.21	2	0	0	172.58
61	Vestitol	-10.7681	3.05	58.92	20	272.3	4	2	2	245.61
62	Isoerysenegalensein E	-10.7655	4.96	111.12	31	422.48	6	4	6	387.33
63	Thunberginol C	-10.7402	2.28	86.99	20	272.26	5	3	1	230.26
64	Eriosematin A	-10.7319	3.27	70.67	18	246.26	4	2	2	221.96
65	(-)-Epiafzelechin	-10.7312	1.86	90.15	20	274.27	5	4	1	236.12
66	Alpinetin	-10.7255	2.66	55.77	20	270.28	4	1	2	239.77
67	Liquiritigenin	-10.7165	2.2	66.76	19	256.26	4	2	1	222.24
68	Naringenin triacetate	-10.7046	1.74	105.22	29	398.37	8	0	7	339.8
69	Isobonducellin	-10.704	3.17	55.77	21	282.3	4	1	2	250.36
70	Naringenin	-10.6376	2.12	86.99	20	272.26	5	3	1	230.26
71	6-Aminoflavone	-10.6331	2.79	56.23	18	237.26	3	2	1	211.28
72	2-Hydroxynaringenin	-10.6282	1.54	107.22	21	288.25	6	4	1	237.96

73	Sulfuretin	-10.6265	1.76	90.89	20	270.24	5	3	1	224.05
74	(+)-Afzelechin	-10.5911	1.86	90.15	20	274.27	5	4	1	236.12
75	5-Acetoxy-7-hydroxyflavone	-10.5609	2.71	76.74	22	296.28	5	1	3	252.54
76	5-Hydroxy-7,8-dimethoxyflavanone	-10.5051	2.92	65	22	300.31	5	1	3	265.32
77	3-O-Acetylpinobanksin	-10.5032	2.38	93.07	23	314.29	6	2	3	266.8
78	Equol	-10.4969	3.07	49.69	18	242.27	3	2	1	220.06
79	5-Hydroxy-7,8-dimethoxyflavanone	-10.4888	2.92	65	22	300.31	5	1	3	265.32
80	Pinobanksin	-10.4823	1.68	86.99	20	272.26	5	3	1	230.29
81	Leachianone A	-10.473	4.28	107.22	26	356.37	6	4	3	315.62
82	4'-Hydroxy-7-methoxyflavan	-10.4695	3.64	38.7	19	256.3	3	1	2	237.59
83	Dihydrobonducellin	-10.4585	3.06	55.77	21	284.31	4	1	3	256.57
84	Galangin	-10.4538	2.65	90.89	20	270.24	5	3	1	224.05
85	Sappanone A	-10.4363	2.15	86.99	21	284.27	5	3	1	240.85
86	Izalpinine	-10.4285	3.19	79.9	21	284.27	5	2	2	241.58
87	Derrone	-10.395	3.95	79.9	25	336.34	5	2	1	291.22
88	3’-Hydroxy-3,9-dihydroeucomin	-10.395	2.26	96.22	23	316.31	6	3	3	272.61
89	Hesperetin	-10.3912	1.94	96.22	22	302.28	6	3	2	255.81
90	Phaseollidin	-10.3904	4.47	58.92	24	324.38	4	2	2	295.57
91	Genkwanin	-10.3809	3	79.9	21	284.27	5	2	2	241.58
92	Demethylvestitol	-10.3749	2.52	69.92	19	258.27	4	3	1	228.08
93	Aromadendrin	-10.3669	1.2	107.22	21	288.25	6	4	1	238.31
94	Glycitein	-10.3463	2.38	79.9	21	284.27	5	2	2	241.58
95	Daidzein	-10.3432	2.56	70.67	19	254.24	4	2	1	216.03
96	DL-Catechin	-10.342	1.37	110.37	21	290.27	6	5	1	244.14
97	Epicatehin	-10.342	1.37	110.37	21	290.27	6	5	1	244.14

98	Morin	-10.34	1.88	131.35	22	302.24	7	5	1	240.08
99	3-Deoxysappanone B	-10.331	2.03	86.99	21	286.28	5	3	2	247.06
100	Butin	-10.3299	1.71	86.99	20	272.26	5	3	1	230.26
101	5,7-Dimethoxyflavanone	-10.3109	3.2	44.77	21	284.31	4	0	3	257.3
102	Quercetin 3-O-beta-D-xylopyranoside	-10.3061	0.06	190.28	31	434.35	11	7	3	347.36
103	2'-Hydroxydaidzein	-10.3022	2.27	90.89	20	270.24	5	3	1	224.05
104	Oroxylin A	-10.3017	2.96	79.9	21	284.27	5	2	2	241.58
105	Dodoviscin I	-10.2984	3.65	120.36	28	386.4	7	4	6	341.41
106	Kurarinol	-10.2964	4.96	116.45	33	456.54	7	4	8	424.98
107	Pelargonidin chloride	-10.2428	-0.26	92.08	20	271.25	5	4	1	226.79
108	Pelargonidin	-10.2428	-0.26	92.08	20	271.25	5	4	1	226.79
109	Kushenol K	-10.2264	4.04	136.68	34	472.53	8	5	8	433.02
110	Viscidulin I	-10.2253	2.12	131.35	22	302.24	7	5	1	240.08
111	Erysubin A	-10.2175	3.44	104.03	26	352.34	6	3	2	299.23
112	Maackiain	-10.1982	2.59	57.16	21	284.27	5	1	0	234.14
113	Tectorigenin	-10.184	2.28	100.13	22	300.27	6	3	2	249.59
114	Sakuranetin	-10.1739	2.65	76	21	286.28	5	2	2	247.79
115	Catechin	-10.1718	1.37	110.37	21	290.27	6	5	1	244.14
116	Erysenegalensein E	-10.1676	4.96	111.12	31	422.48	6	4	6	387.33
117	Dihydrooroxylin A	-10.1626	2.4	76	21	286.28	5	2	2	247.79
118	Medicarpin	-10.1432	2.76	47.93	20	270.28	4	1	1	235.76
119	Luteolin	-10.1259	1.97	111.12	21	286.24	6	4	1	232.07
120	Robtin	-10.0583	1.42	107.22	21	288.25	6	4	1	238.28
121	Dihydrorobinetin	-10.0583	0.5	127.44	22	304.25	7	5	1	246.32
122	Kaempferol	-10.0098	2.17	111.12	21	286.24	6	4	1	232.07

Supplementary Figure 1: a) Best docking conformation of Isobavachin and 1E3G (Binding affinity: -13.7368 kcal/mol) ; b) Best docking conformation of Glabranin and 1E3G (Binding affinity: -13.2674 kcal/mol); c) Best docking conformation of Anthocyanin and 1E3G (Binding affinity: -13.0199 kcal/mol); d) Best docking conformation of Eriosemation and 1E3G (Binding affinity: -12.7852 kcal/mol).

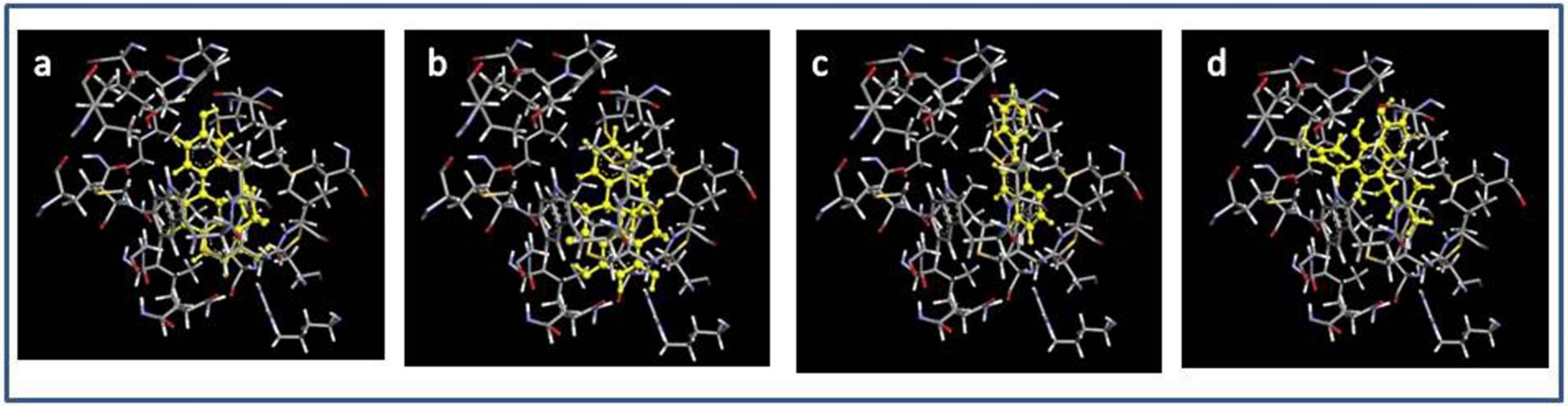

Supplement: Supplementary file 1 — Supplementary Information [file 41598_2017_2023_MOESM1_ESM.pdf]
